# The effect of negative emotion processing on spatial navigation: an experimental study using virtual reality

**DOI:** 10.3389/fpsyg.2023.1301981

**Published:** 2024-01-11

**Authors:** Linda Mohamed Aly, Matteo Masi, Massimo Montanaro, Paola Ricciardelli

**Affiliations:** ^1^Department of Psychology, University of Milano-Bicocca, Milan, Italy; ^2^Faculty of Health and Medical Sciences, University of Surrey, Guildford, United Kingdom; ^3^MiBTec, University of Milano-Bicocca, Milan, Italy; ^4^Department of Psychology, University of Münster, Münster, Germany; ^5^NeuroMI, Milan Center for Neuroscience, Milan, Italy

**Keywords:** spatial navigation, faces, emotions, fear, virtual reality

## Abstract

Finding one’s way in unfamiliar environments is an essential ability. When navigating, people are overwhelmed with an enormous amount of information. However, some information might be more relevant than others. Despite the mounting knowledge about the mechanisms underlying orientational skills, and the notable effects of facial emotions on human behavior, little is known about emotions’ effects on spatial navigation. Hereby, this study aimed to explore how exposure to others’ negative emotional facial expressions affects wayfinding performances. Moreover, gender differences that characterize both processes were considered. Fifty-five participants (31 females) entered twice in three realistic virtual reality environments: the first time, to encode a route to find an object and then to recall the learned path to reach the same object again. In between the two explorations of the virtual environment, participants were asked to undergo a gender categorization task during which they were exposed to sixty faces showing either neutral, fearful, or angry expressions. Results showed a significant interaction between emotions, time, and gender. In particular, the exposition to fearful faces, but not angry and neutral ones, decreased males’ wayfinding performances (i.e., travel times and distance travelled), while females’ performances were unaffected. Possible explanations for such gender and emotional dissimilarities are discussed.

## Introduction

Navigating unfamiliar environments requires prioritizing continuous awareness of one’s location relative to the surroundings to avoid getting lost. This ability is vital for situational awareness, planning and preparedness for action (Smith and Hancock, 2020). Meanwhile, we are influenced by a considerable amount of information, but some might be more relevant than others in affecting navigation. Despite the increasing number of studies regarding spatial navigation (Burgess, 2008; Wolbers, 2015; Epstein et al., 2017; Ekstrom et al., 2018), little is known about the effect of processing facial expressions on these dynamics. Similarly, emotion research overlooked the influence of processing of others’ facial emotions on navigation behavioral performances (Phelps, 2006; Pessoa, 2009; Burles et al., 2020). Hence, in this research, we aimed to uncover whether processing others’ negative facial emotional expressions affect spatial navigation in virtual reality.

Spatial navigation is the process underlying the ability to orient oneself in a familiar or new environment, enabling travelling in the real world (Brown and Chrastil, 2019; Diersch and Wolbers, 2019). It requires a wide range of cognitive abilities, including attention, memory, decision-making, and problem-solving (O’keefe and Nadel, 1978; Brodbeck and Tanninen, 2012). In this research we focus on wayfinding behaviors (see Golledge, 1999; Farr et al., 2012; Wolbers, 2015), that is the ability to locate oneself in space using multiple sources of cues to determine the path to a destination and then travel to it (Ekstrom et al., 2018). Body-based self-motion cues and environmental information are integrated over time for wayfinding to be effective (Sjolund et al., 2018). As a higher-order function, it requires abilities to use different references in the space (i.e., allocentric, egocentric), multiple integration processes (i.e., visual, proprioceptive, vestibular) and knowledge-based strategies (i.e., landmark, survey, route; Van der Ham et al., 2020). Altogether, these mechanisms allow estimating directions, learning positions, adjusting errors, reaching locations, and remembering destinations, ensuring an effective navigation (Siegel and White, 1975; Montello and Raubal, 2013). Behavioral indicators of wayfinding performances can be the time it takes to reach a destination and the distances travelled while searching for it (Saucier et al., 2002; Coluccia and Louse, 2004; Burke et al., 2012; Dong et al., 2022).

While navigating an environment, people are often exposed to information among which they filter out those that are irrelevant to the task at hand (e.g., advertising hoardings, flashing lights, and sudden sounds; Kunishige et al., 2020; Stangl et al., 2020). However, some information might be evolutionarily, psychologically, and socially more relevant than others: one clear-cut example is others’ facial emotions (Ekman, 1993). Emotional faces are stimuli able to communicate positive and affiliative affects, but also negative and arousing ones (Marsh et al., 2005). As part of everyday social life, people recognize faces in the environment and identify their emotional expressions and the spatial location in which they were seen with precision and haste (White and Burton, 2022). While walking on the street or entering a building, it is often the case to look at another person’s facial expression to understand whether our own behavior is appropriate to the situation surrounding us. Indeed, through facial expressivity people communicate considerable information relevant for managing social situations (Langfeld, 1918).

From a cognitive perspective, emotions are pervasive cues that influence, among the many human cognitive functions, spatial cognition and orientation (Schupp et al., 2003; Pourtois et al., 2004; Bisby and Burgess, 2014). For example, Ruotolo et al. (2019) tested the effect of emotional landmarks on a series of spatial memory tasks. They found that the position of positive landmarks is remembered more accurately than neutral and negative landmarks’ position, but routes with negative landmarks are remembered as longer to travel than those with the other landmarks (see also Piccardi et al., 2020; Rasse et al., 2023). Such study is one of the few showing that spatial memory, one of the functions necessary to wayfinding (Van der Ham et al., 2020), can be influenced by emotional cues. However, testing the effect of emotion elicited by emotional objects (e.g., dogs, books, guns) on spatial memory is not the same as testing the effect of emotions perceived from faces specifically, much less when it comes to wayfinding behaviors in realistic settings. In wayfinding research, the critical role of social interactions, often driven by emotions, has been highlighted as a potential influencer of decision-making during navigation (Dalton et al., 2019).

To the best of our knowledge the consequences of the exposure to other people’s emotions for wayfinding behaviors have been hardly investigated and, therefore, an examination of the consequences is needed to start filling such a gap.

Our interest is directed to the effect of negative facial expressions (i.e., fear and anger). There is consensus among researchers that they significantly impact people’s behaviors more than neutral or positive ones (Stins et al., 2011). The ability of communicating and perceiving emotions is thought to represent a substantial adaptive advantage for humans and animals for predicting other individuals’ future actions and adjusting one’s own behavior accordingly (González-Garrido et al., 2013). Evolutionarily, peoples’ attention to such facial cues comes from their ability to discern threats for survival advantage, even when the precise nature of the threat remains only partially understood (Adolphs, 2008). In fact, negative emotions are supposed to be quickly and effectively recognized with the aim of activating motor reactions (e.g., fight/flight, Öhman et al., 2001). However, such motor reaction differs according to the perceived emotion. For instance, research suggested that perceiving other’s fear, which might inform about a threat source in the surroundings (i.e., someone is chased by a dangerous animal), can lead to approach behaviors towards conspecifics to help; instead, perceiving other’s anger, which might signal another person’s intent to aggress, can push towards avoidance behaviors to escape the immediate confrontation (Marsh et al., 2005). However, not all findings are concordant: Adams et al. (2006) suggested that fearful faces might instead elicit freezing responses (i.e., behavioral inhibition; but see Bossuyt et al., 2014). Additionally, encountering a fearful face during a response inhibition task has been shown to enhance the ability to inhibit a motor response (Choi and Cho, 2020). Mirabella (2018), using a Go/No-Go task, showed that fearful faces increase the error rates and reaction times more than happy faces and Mancini et al. (2022) indicated that fearful faces enhanced inhibitory control compared to happy faces, but only if emotions were relevant to the task (see Mancini et al., 2020 for a comparison with angry faces; see also Mirabella et al., 2023). Interestingly, the perceived contrast between fear and anger (and other emotions) can also influence behavioral reactions: when fearful and angry expression are presented in the same task and there is no comparison with a positive emotion (i.e., happiness), anger leads to approach and fear to avoidance, but both lead to avoidance when presented together with positive emotions (Paulus and Wentura, 2016). According to these findings, the studies on whether and how the processing of threatening emotions can affect behavioral reactions of people have led to mixed results.

Gender differences should be considered when studying navigation (Fischer et al., 2018; Munion et al., 2019; Olderbak et al., 2019). In general, males are better at wayfinding than females (Coluccia and Louse, 2004; Clint et al., 2012). Potential factors contributing to gender differences in spatial memory include biological differences, such as right hemisphere dominance and higher levels of the hormone testosterone in males (Driscoll et al., 2005; Persson et al., 2013), or environmental factors, such as the amount of time spent playing video games with a strong spatial component (Baenninger and Newcombe, 1989). In addition, researchers suggest that gender differences in opportunities to explore new environments may also play a role: in some cultural environments, boys might be allowed more than girls to explore new environments (Webley, 1981). Indeed, a combination of these factors may exacerbate gender differences in spatial and navigational skills (Casey, 1996; Clements et al., 2006; Voyer et al., 2007). In addition, a review by Coluccia and Louse (2004) showed that spatial anxiety, which has a significant impact on navigation, may differ between the genders, with females exhibiting higher spatial anxiety than males (Lawton, 1994).

Similarly, gender differences are frequently observed in emotion processing. On one hand, studies found that females might be better at recognizing emotions from facial expressions (Montagne et al., 2005; Kret and De Gelder, 2012) even when subtly expressed (Hoffmann et al., 2010), although recent evidence on a large sample is discordant with this latter finding (Fischer et al., 2018). On the other hand, males show greater behavioral responses to threatening cues than females, possibly explained by diverse motor tendencies (Han et al., 2008; Kret and De Gelder, 2012). At the brain level, men showed a greater amygdala activation for threatening scenes than females, a brain area often responsive to threatening cues (Schienle et al., 2005). Therefore, it might be reasonable to expect gender differences both in spatial navigation and in the influence that emotion processing has on it.

## The present research

In the present study, we immersed participants in a simulated environment that represents a moderately ecological way to investigate wayfinding behaviors. Virtual reality (VR) could be crucial in assessing wayfinding performances (Jeung et al., 2023) owing to its capacity to replicate immersive environments, facilitate natural movements, and enable navigation with a heightened sense of presence that provides an almost natural field of view. It captures the dynamic nature of navigation, presenting an ecological environment with control over behavioral measures. Additionally, research indicates that cognitive maps and representations of large-scale spaces in virtual reality are similar to those obtained in a natural environment (Ruddle et al., 1999).

In a VR environment (i.e., an office building with multiple floors), participants were introduced to a wayfinding task consisting of first finding an object located in the environment (i.e., encoding phase, T1) and subsequently finding the same object at the same location (i.e., recalling phase, T2). Emotional faces showing fearful, angry, or neutral expressions were shown between the two phases in a task unrelated to wayfinding (i.e., gender categorization). We measured travel times and distances travelled at T1 and T2 as behavioral outcomes related to navigation performances of the participants (Burke et al., 2012; Dong et al., 2022) and we investigated whether there were any differences due to the emotional conditions and gender of participants.

Our research question concerned whether exposure to others’ facial expressions could facilitate or limit navigational performance during a wayfinding task. First, we expected participants to show faster travel times and shorter distance travelled in the recalling phase than in the encoding phase due to learning and familiarity after the exploration. Second, based on our review of the available research, we expected that the exposure to a threatening emotional stimulus (i.e., fearful or angry face) might interfere with wayfinding performances. Threatening emotional faces might affect behavioral tendencies of participants asked to navigate an environment, potentially moderating the outcome of wayfinding performances. However, due to the novelty of our investigation, it was not possible to precisely hypothesize about the direction of the effect, that is whether threatening faces might improve wayfinding performances or impair them, and whether fear or anger differed in their effect. We compared their effects and provided a potential explanation for the pattern of results in the discussion section. Moreover, gender differences were expected to modulate spatial navigation and the effect of processing negative facial emotions on it.

We assumed that facial expressions of emotion could impact the subsequent recall phase even when participants were not explicitly instructed to focus on such stimuli during the primary wayfinding task. There is evidence that threatening stimuli can affect the allocation of attentional resources, even when they are not presented as essential components of cognitive and behavioral tasks (Paulus and Wentura, 2016; Zsidó et al., 2022, 2023) particularly in situations of high cognitive demand (Pessoa et al., 2012). Collectively, we based our assumptions on the possibility that such stimuli can impact the outcomes of tasks even when participants are not directly expected to pay attention to the emotion expressed by faces (Chen and Bargh, 1999; Pessoa, 2009; O’Toole et al., 2011; Ricciardelli et al., 2012; Berggren and Derakshan, 2013; Paulus and Wentura, 2016; Celeghin et al., 2020; Zsidó et al., 2023). It is worth noting, however, that there are contrasting findings in this regard (Berger et al., 2017; Mancini et al., 2020, 2022; Mirabella et al., 2023; see also our discussion section).

## Methods

### Participants

For the present study we collected a sample of 58 healthy student participants using the university recruitment website and snowball sampling^1^. Data collection took place in part during the Covid-19 restrictions in Italy (2020–2021). Since we could not base our sample estimation on a known target effect size, due to the novelty of the design, we did not run *a priori* power analysis and collected participants for 6 months. We limited the age to a range between 18 and 40 years to avoid the natural decline in navigation functionality and limit the side effects of cybersickness (Lithfous et al., 2014; Diersch and Wolbers, 2019). Participants with vision disparities not corrected to normal vision, suffering from neurological conditions (e.g., epilepsy), and/or sea/car sickness, who might be sensitive to virtual reality side effects, were not included in the study. Three participants dropped out during the experiment due to cybersickness and were excluded from the analysis. Our final sample was consisting of 55 participants (24 males, *M_age_* = 23.5, *SD_age_* = 2.72; 31 females, *M_age_* = 21.5, *SD_age_* = 2.55). We ran a sensitivity power analysis which showed that our study could detect a minimal effect of *η*^2^_p_ = 0.08 (Cohen’s *f* = 0.30) with this sample size, and power = 0.80 at α = 0.05 (Campbell and Thompson, 2012).

The study was approved by the Committee for Research Evaluation (CRIP) of the Department of Psychology of the University of Milan-Bicocca (RM 2020-366). All participants received written informed consent and were treated in accordance with the Declaration of Helsinki. Participants received university credits in exchange for their participation.

### Apparatus

**Figure 1:**
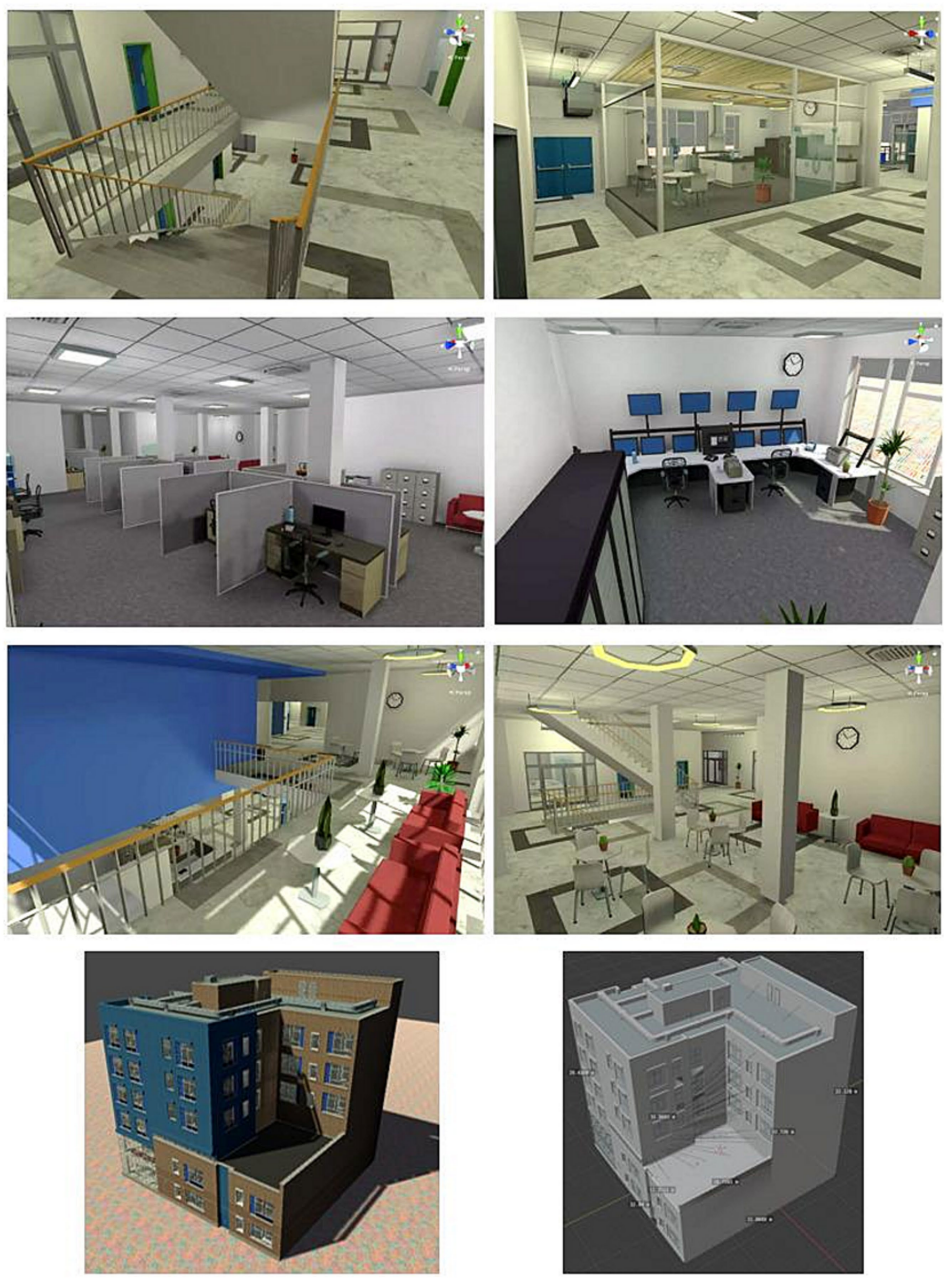
Images of the office building asset created in unity. The asset was retrieved from the unity asset store (255 Pixel Studios (2018), Jun 21, 2018).

The Oculus Rift was utilized to project the entire experiment to the participants. The head-mounted device featured a 1,280 × 1,440 LCD with an 80 Hz refresh rate and a field of view measuring 86° × 86°. During navigation sessions, participants had autonomous control over their movements using two controllers. For the navigational task, a customized “office building” consisting of four floors was created (see Figure 1). Floor 0 was used for training, while Floors 1, 2, and 3 were used for testing. The mazes within the environment were designed and configured using the Unity cross-platform game engine. Each floor contained various barriers within an enclosed arena, with no written indications. Distinct pieces of furniture served as landmarks or reference points for participants, which were repeated on each floor. Stair access and elevator usage were not permitted. Participants had a standard speed of 2 “unity meters” per second, but they could adjust their speed by ±0.5 meters using the controller’s buttons. This setup allowed participants to choose their preferred speed at all stages and mitigate cybersickness.

For each condition (three in total), the encoding phase and the recalling phase took place on the same floor to ensure comparable performances before and after the exposure to emotional stimuli. Therefore, each participant entered each floor twice for being exposed once to each of the three emotional expressions. The starting point and target object were always in the same position on each floor but differed between floors. The order of floor presentation and assignment of emotional conditions were counterbalanced. Additionally, slight variations in the maps of the floors were introduced to avoid repetition of the map conformation (see below the test of maps’ heterogeneity).

Following Nazareth et al. (2019) coding scheme our wayfinding task has the following features: environment: indoor (office); testing medium: VR (Oculus Rift-S); route perspective: route (first-person walking, no teleportation); route selection: free choice-not taught; timing conditions: limited (10 min maximum per session); cues: proximal (non-interactive landmarks); familiarity: learned; feedback: immediate (target location found in each trial); hints: no helping provided; device assistance: not present; learning interval: immediate (testing begins after manipulation); outcome measures: times and distances (seconds and Unity’s meter unities).

### Materials

#### Stimuli

**Figure 2:**
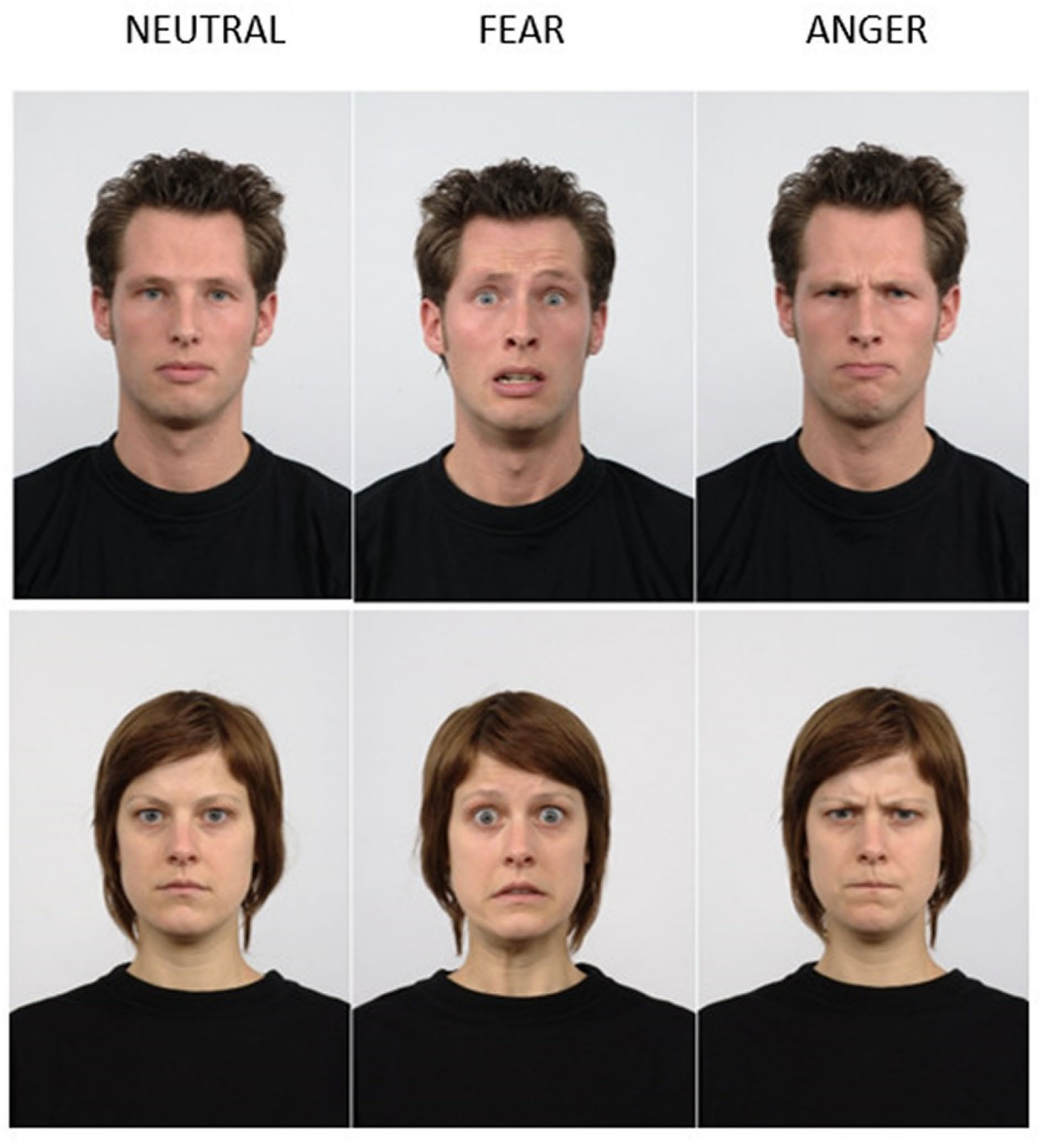
Examples of male and female expressing fearful, angry, and neutral faces extracted from the Radboud Face Database (Langner et al., 2010). The list of stimuli id used extracted from the Rafd are available on OSF: https://osf.io/wzbvy/. See Radboud Faces Database (ru.nl) to have access to the database.

The faces used for the emotion categorization task were selected from the Radboud Faces Database (Langner et al., 2010). The dataset consisted of 15 female and 15 male frontal faces expressing neutral, fearful, or angry expressions (see Figure 2).

### Procedure

**Figure 3:**
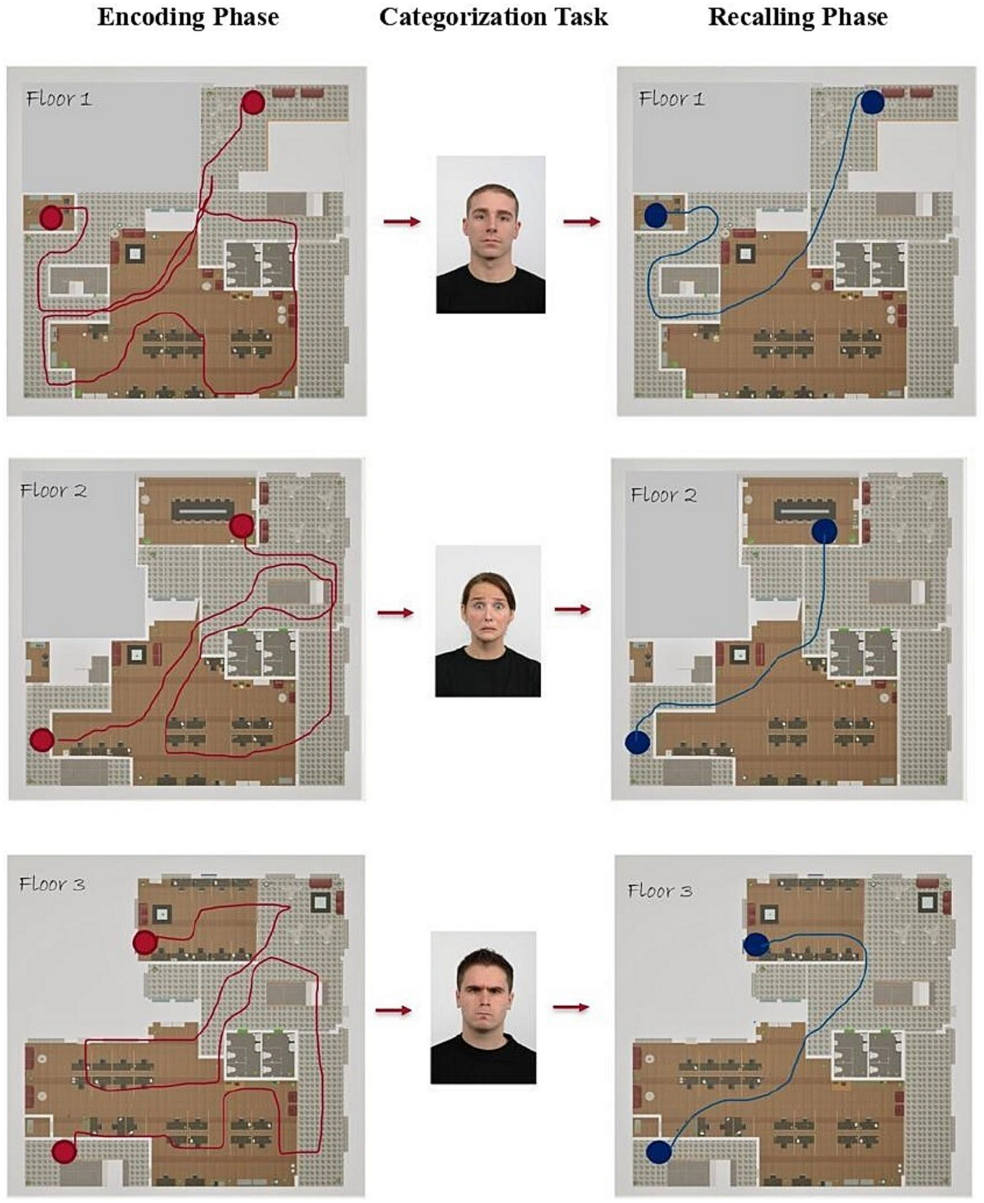
Example of the procedural sequence with footprints. Encoding phases are shown on the left, the categorization task in the middle, and the recalling phases on the right.

Participants were introduced to the laboratory, signed the informed consent, and received formal instructions about the experimental phases: training, encoding, categorization, and recalling (see Figure 3 for a schematic example of the procedure).

#### Training

We asked participants to put on the Oculus Rift HMD and enter the practice floor for five minutes to familiarize themselves with the setting, the task, and the target object (i.e., blue box). Habituation to the tool was intended to reduce predictable cybersickness symptoms (e.g., headache, blurred vision, motion sickness, nausea). The training floor differed from those used in the testing so as not to affect the main task’s results. At the end of the training, participants began the experimental phases, which were two (encode vs. recall) for each of the three emotional conditions.

#### Encoding phase

Participants entered a new floor randomly picked among a set of three (counterbalanced between subjects) and were instructed to explore the environment to find the target object, always a blue box. We reminded them to pay attention to the surroundings and remember the route taken to get to the object. Once they found the object, they started the next phase.

#### Categorization task

**Figure 4:**
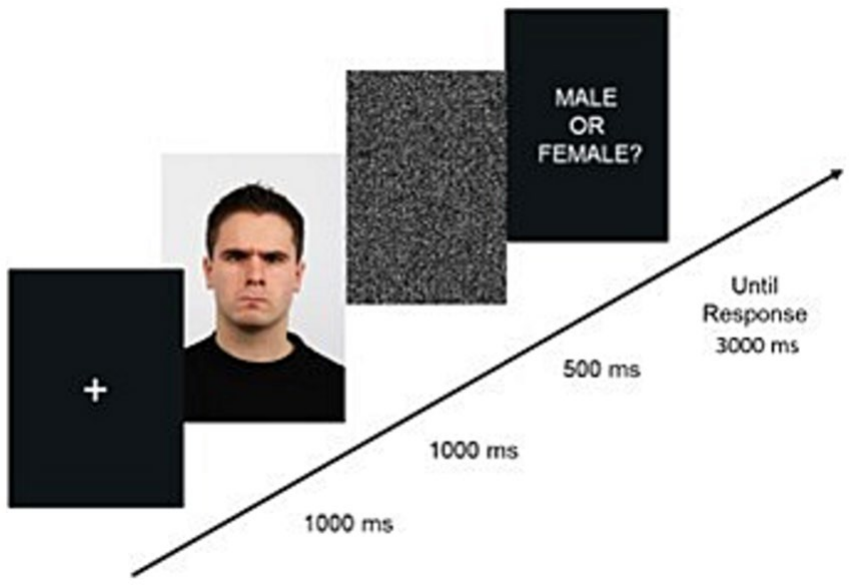
Example of the sequence of events in a trial for the categorization task.

Within the same virtual setting and after a short break time, participants entered a grey-walled bright room. They were asked to take part in a categorization task based on face stimuli. Instructions told them to make the responses using the controller’s buttons at they own pace. We told them to take their time in answering because the main goal of the procedure was the prolonged exposure to the emotional stimuli. During such task they were shown a series of face pictures projected on the wall in front of them. The faces’ dimensions were kept as close as possible to those seen on a PC monitor with a viewing distance of about 50 cm. The faces expressed neutral or fearful or angry emotions, depending on the conditions, which were counterbalanced between-participants. The face stimuli were repeated twice in random order per task (60 stimuli in total). The trial consisted of: a fixation cross for 1,000 msec; the emotional face for 1,000 msec; a mask for 500 msec; a question asking “Male [Female] or Female [Male]?” with labels and button responses counterbalanced between participants. At the end of the trial, we allowed a maximum response time of 3,000 msec. The task lasted on average *circa* 5 min (see Figure 4 for a graphical representation).

#### Recalling phase

Participants immediately returned to the same floor of the encoding phase to test their ability to find again the box which was placed in the same location of the encoding phase.

We eventually measured different exploratory variables which descriptive results are reported in Supplementary material. Finally, participants were thanked and debriefed.

### Data preparation and statistical analyses

We employed a 3 (emotion: neutral vs. fearful vs. angry faces) × 2 (time: encoding vs. recalling) × 2 (gender: female vs. male) mixed subjects design, with gender as a between-subjects factor.

As dependent variables, we recorded travel times in milliseconds from the moment they entered each floor until they reached the target object. Moreover, we measured the distances travelled from the first step until reaching the object using a Euclidean formula for calculating the distance between one temporally ordered position and the following one, then we summed the results. The latter scores were based on participants’ x and z coordinates on the floor registered five times per second. The distances are expressed with an internal Unity’s unit of measure (um). For both travel times and distance travelled we created average scores for each experimental phase. Analyses were carried out on Jamovi (2023) and R Core Team (2021).

For the analyses, we first checked whether our data respected the assumptions of parametric tests. For testing the normality of errors, we inspected the QQ-plot of the fitted models’ residuals. For the homogeneity of variance, we conducted Leven’s tests. For the sphericity assumption, we conducted Mauchly’s tests. For both the dependent variables, the assumptions were not respected (see Supplementary material). Therefore, we applied a logarithmic transformation to the two dependent variables to obtain pseudo-normal data (Marmolejo-Ramos et al., 2015). The transformation shifted and centralized the extremities, reducing the impact of extreme observations. After transformation, the assumptions were not violated anymore. Hence, we proceed with conducting mixed ANOVAs. For each analysis, for sake of simplicity and to highlight the differences between the three emotional conditions, in examining the interactions between emotion and time (i.e., two-way interaction), and between these factors and gender (i.e., three-way interaction), we calculated a differential score by subtracting Time 2 performance from Time 1 (T1-T2) within each emotional condition. The higher the score, the better the performances at T2 compared with T1. Simple effects analyses were adjusted with the Bonferroni-Holm method. We reported effect sizes (*η*^2^_p_ for the *F* tests, *d* for the *t* tests) along with the statistical tests (Cohen’s *d* are reported in absolute value for easier interpretation).

We also made preliminary analyses to test whether the travel time and the distance travelled on each floor was balanced and not dependent on the heterogeneity of the floor maps. A repeated-measures ANOVA comparing the three floors performed on T1 only – as the emotional exposure had not yet been delivered – showed that the three maps produced no differences in time spent in each floor, *F*(2,106) = 0.57, *p* = 0.56, *η*^2^_p_ = 0.011 (Floor 1: *M* = 4.56, *SD* = 0.59; Floor 2: *M* = 4.49, *SD* = 0.67; Floor 3: *M* = 4.59, *SD* = 0.59, see Table 1). While the main effect of gender was significant, *F*(1,53) = 19.0, *p* < 0.001, *η*^2^_p_ = 0.26, as males spend on average less time on each floor than females, the interaction between maps and gender was non-significant, *F*(2,106) = 0.23, *p* = 0.79, *η*^2^_p_ = 0.004. The same analysis on distance travelled showed no differences between floors, *F*(2,106) = 0.67, *p* = 0.52, *η*^2^_p_ = 0.012 (Floor 1: *M* = 4.87, *SD* = 0.48; Floor 2: *M* = 4.78, *SD* = 0.51; Floor 3: *M* = 4.80, *SD* = 0.05; see Table 1). The main effect of gender was again significant, *F*(1,53) = 7.09, *p* = 0.01, *η*^2^_p_ = 0.12, while the interaction between maps and gender was non-significant, *F*(2,106) = 0.58, *p* = 0.56, *η*^2^_p_ = 0.01. Therefore, we can conclude that maps were homogenous in their times and distances travelled.

**Table 1:** Means and standard deviations (in parenthesis) of travel time and distance travelled in seconds after the logarithmic transformation at T1 – before the exposure to emotions – as a function of the gender of the participants.

Map	Travel time		Distance travelled	
	Female	Male	Female	Male
Floor 1	4.77 (0.56)	4.28 (0.51)	4.95 (0.46)	4.78 (0.50)
Floor 2	4.73 (0.59)	4.19 (0.65)	4.93 (0.45)	4.60 (0.54)
Floor 3	4.76 (0.56)	4.36 (0.55)	4.86 (0.51)	4.71 (0.51)

## Results

### Analysis on travel time

To determine whether participants’ performances were affected by the emotions presented in the emotional conditions, we performed a 3 (emotion: neutral vs. fearful vs. angry faces) × 2 (gender: female vs. male) × 2 (time: T1 vs. T2) mixed ANOVA on travel times. Means and standard deviations are reported in Table 2.

**Table 2:** Means and standard deviations (in parenthesis) of travel time in seconds as a function of gender and emotional conditions with and without the logarithmic transformation.

Times (sec)	Female			Male		
Emotion	T1	T2	T1–T2	T1	T2	T1–T2
Neutral	148 (112.6)	56.55 (33.4)	91.61 (118.6)	100 (71.6)	34.7 (22.6)	65.4 (78.1)
Fear	152 (108.6)	54.77 (44.5)	97.39 (105.3)	68.3 (35.0)	59.8 (36.8)	8.46 (57.5)
Anger	113 (58.1)	58.55 (56.3)	55.19 (64.3)	86.5 (46.5)	38.6 (17.6)	47.9 (47.0)
**Times (log)**						
Neutral	4.82 (0.55)	3.88 (0.55)	0.93 (0.74)	4.40 (0.60)	3.40 (0.51)	1.00 (0.86)
Fear	4.82 (0.64)	3.80 (0.61)	1.02 (0.71)	4.08 (0.57)	3.90 (0.62)	0.17 (0.95)
Anger	4.61 (58.1)	3.81 (0.66)	0.80 (0.65)	4.34 (0.47)	3.56 (0.41)	0.77 (0.55)

The results showed a significant main effect of time, *F*(1,53) = 167.06, *p* < 0.001, *η*^2^_p_ = 0.77, such that participants were faster at T2 than T1 showing that they learned the route and recalled it effectively, and a significant main effect of gender, *F*(1,53) = 15.4, *p* < 0.001, *η*^2^_p_ = 0.22, with males on average being faster than females in reaching the object. The main effect of emotion was not significant, *F*(2,106) = 0.55, *p* = 0.58, *η*^2^_p_ = 0.01, as well as the interaction between gender and emotions, *F*(2,106) = 1.10, *p* = 0.34, *η*^2^_p_ = 0.02. The interaction between gender and time was significant, *F*(1,53) = 4.88, *p* = 0.03, *η*^2^_p_ = 0.08. A simple effects analysis showed that, while at T1 males were faster than females, *t*(94.7) = 4.47, *p* < 0.001, *d* = 0.46, the difference with females was reduced at T2, *t*(94.7) = 1.95, *p* = 0.05, *d* = 0.20. Importantly, the interaction between emotion and time was significant, *F*(2,106) = 3.37, *p* = 0.04, *η*^2^_p_ = 0.04. Decomposing the interaction revealed that there was no difference in times between the three emotions at T1, *t*(211) < |1.63|, *p*s > 0.31, *d* < 0.11, and at T2, *t*(211) < |2.16|, *p*s > 0.09, *d* < 0.15. However, this interaction is better inspected with a simple effects analysis on the differential score (T1–T2) within each emotion condition, which showed that participants were slower at T2 compared to T1 after the fearful condition compared to the neutral one, *t*(106) = −2.59, *p* = 0.03, *d* = 0.25, while no other comparisons were significant, *t*(106) < 1.35, *p* > 0.36, *d* < 0.13.

**Figure 5:**
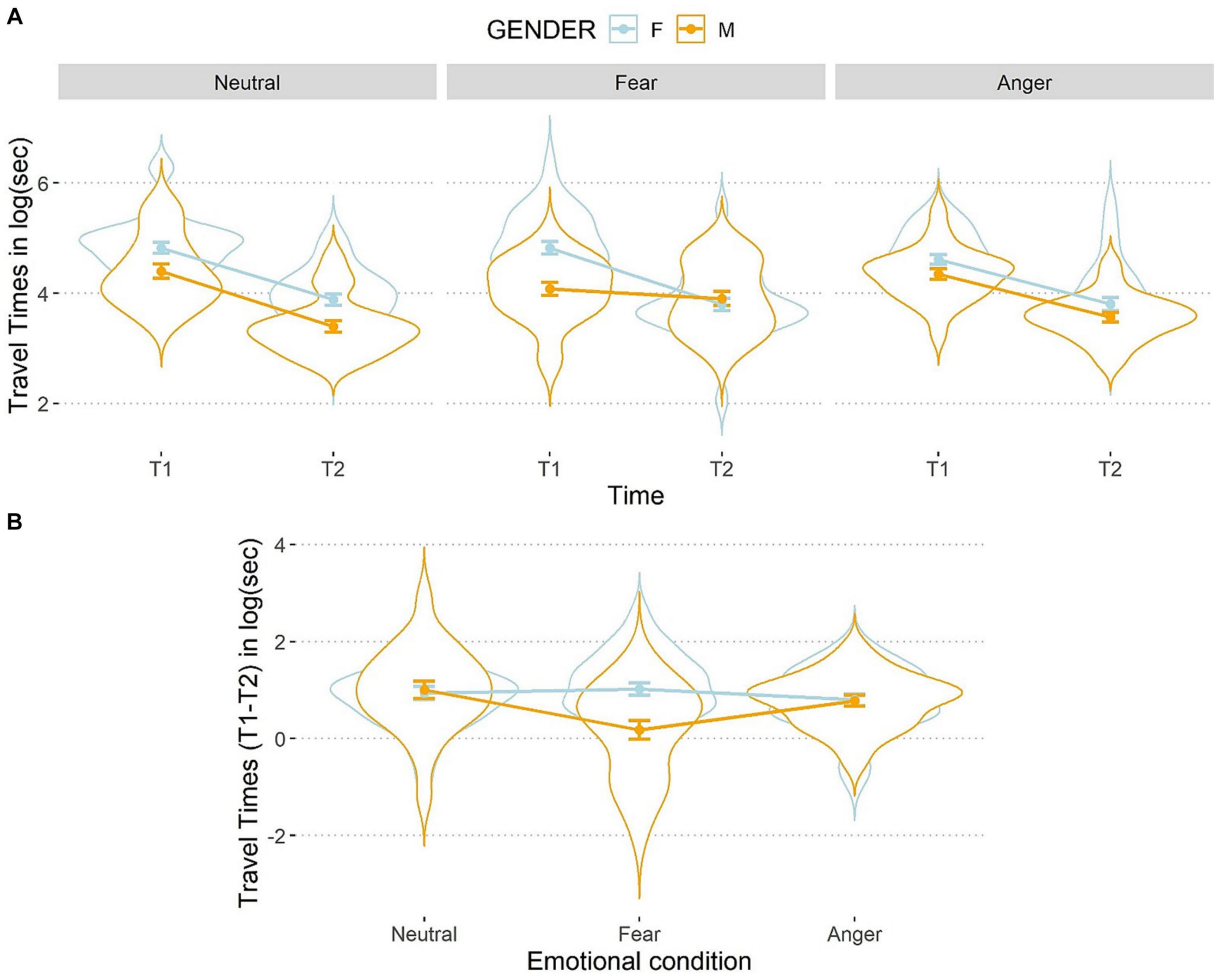
Line and violin plots representing the difference in travel times in seconds (after the logarithmic transformation) between single encoding (T1) and recalling (T2) sessions **(A)** and differential score (T1–T2) **(B)** as a function of gender and emotional conditions. Lower numbers indicate better performances in graph a, and vice versa in graph b. The light blue line represents female participants, and the orange line represents male participants. Bars represent standard error around the means.

The three-way interaction between emotions, time, and gender was significant, *F*(2,106) = 6.12, *p* = 0.003, *η*^2^_p_ = 0.10 (see Figure 5).

Participants improved their performances from T1 to T2 in all conditions, *t*(159) < 7.53, *p* < 0.001, *d* < 0.60, except for males who did not exhibit any improvement after being exposed to the fearful condition reflected in a non-significant difference between T1 and T2, *t*(159) = 1.14, *p* = 0.25, *d* = 0.09. At T1, a simple effects analysis on females showed no differences between the three emotional conditions, *t*(211) < 1.79, *p*s > 0.50, *d* < 0.09, as well as for males, *t*(211) < 1.79, *p*s > 0.09, *d* < 0.16, showing that baseline performances were balanced between emotional conditions. At T2, females did not perform differently according to the emotional conditions, *t*(211) < |0.31|, *p*s > 0.99, *d* < 0.04. Conversely, at T2 males showed a significant difference between the angry and the fearful, *t*(211) = −2.30, *p* = 0.04, *d* = 0.16, as well as between the fearful and the neutral conditions, *t*(211) = 3.43, *p* < 0.002, *d* = 0.24. This suggests that males were slower after being exposed to fearful faces than after the other two types of faces. Interestingly, no significant difference was found between the anger and the neutral conditions, *t*(211) = 1.13, *p* = 0.26, *d* = 0.08.

To better inspect this three-way interaction, we analyzed the differential score (T1–T2). For females, the travel times did not differ between the three emotion conditions, *t*(106) < |1.14|, *p*s > 0.76, *d* < 0.11, while males were slower at T2 than T1 after being exposed to fearful compared to angry, *t*(106) = 2.80, *p* = 0.01, *d* = 0.27, and to neutral faces, *t*(106) = −3.84, *p* < 0.001, *d* = 0.37, but no difference emerged for males between the anger and neutral conditions, *t*(106) = 1.04, *p* = 0.30, *d* = 0.10. The comparison between the two genders within each emotional condition showed that, in the fearful condition, males were significantly slower than females, *t*(159) = 4.11, *p* < 0.001, *d* = 0.65. However, no such a difference emerged in the anger, *t*(159) = 0.12, *p* = 0.89, *d* = 0.02, or in the neutral conditions, *t*(159) = −0.30, *p* = 0.76, *d* = 0.05. Hence, the present results are in line with the conclusion that fear was disrupting males’ performance more than the other emotions. Such a result was not mirrored on females which performances were not influenced by any condition.

### Analysis of distance travelled

We performed a mixed ANOVA 3 (emotion: neutral faces vs. fearful faces vs. angry faces) × 2 (gender: female vs. male) × 2 (time: T1 vs. T2) on distance travelled. Means and standard deviations are reported in Table 3.

**Table 3:** Means and standard deviations (in parenthesis) of distance travelled in seconds as a function of gender and emotional conditions with and without the logarithmic transformation.

Distance (um)	Female			Male		
Emotion	T1	T2	T1–T2	T1	T2	T1–T2
Neutral	154 (83.2)	81.6 (46.2)	72.9 (99.7)	135 (80.0)	63.1 (39.7)	71.5 (94.9)
Fear	152 (83.7)	75.7 (50.2)	75.9 (91.5)	102 (49.3)	112 (68.1)	−9.70 (91.8)
Anger	114 (61.1)	80.7 (64.5)	63 (72.0)	138 (76.0)	78.4 (47.5)	59.8 (99.8)
**Distance (log)**						
Neutral	4.97 (0.45)	4.27 (0.49)	0.69 (0.73)	4.77 (0.51)	4.02 (0.46)	0.75 (0.75)
Fear	4.86 (0.58)	4.18 (0.50)	0.68 (0.66)	4.52 (0.47)	4.54 (0.60)	−0.02 (0.83)
Anger	4.87 (0.41)	4.22 (0.53)	0.65 (0.59)	4.70 (0.53)	4.24 (0.46)	0.55 (0.77)

The main effect of time was significant, *F*(1,53) = 88.48, *p* < 0.001, *η*^2^_p_ = 0.62, indicating that the distances travelled were inferior at T2 than T1. The main effects of gender, *F*(1,53) = 1.70, *p* = 0.20, *η*^2^_p_ = 0.03, and emotions, *F*(1,53) = 0.73, *p* = 0.40, *η*^2^_p_ < 0.001, were not significant. The interaction between time and gender was significant, *F*(1,53) = 4.53, *p* = 0.04, *η*^2^_p_ = 0.08. A simple effects analysis showed that, while at T1 males’ travelled distances were shorter than females, *t*(106) = 2.42, *p* = 0.02, *d* = 0.24. However, this difference with females was canceled out at T2, *t*(106) = −0.51, *p* = 0.61, *d* = 0.05. The interaction between gender and emotions was not significant, *F*(2,106) = 2.00, *p* = 0.14, *η*^2^_p_ = 0.04, whereas the interaction between emotions and time was significant, *F*(2,106) = 4.44, *p* = 0.01, *η*^2^_p_ = 0.08. A simple effects analysis examining the interaction revealed that there was no difference in distance travelled between the three emotions at T1, *t*(211) < |1.93|, *p*s > 0.16, *d* < 0.13, and at T2, *t*(211) < |2.29|, *p*s > 0.07, *d* < 0.16. In addition, this interaction can be further inspected with a simple effects analysis on the differential score (T1–T2) within each emotional condition showing that participants travelled longer distances at T2 compared to T1 after the fearful condition compared to the neutral one, *t*(106) = −2.91, *p* = 0.01, *d* = 0.28, and no other comparisons were significant, *t*(106) < 2.01, *p*s > 0.09, *d* < 0.20.

**Figure 6:**
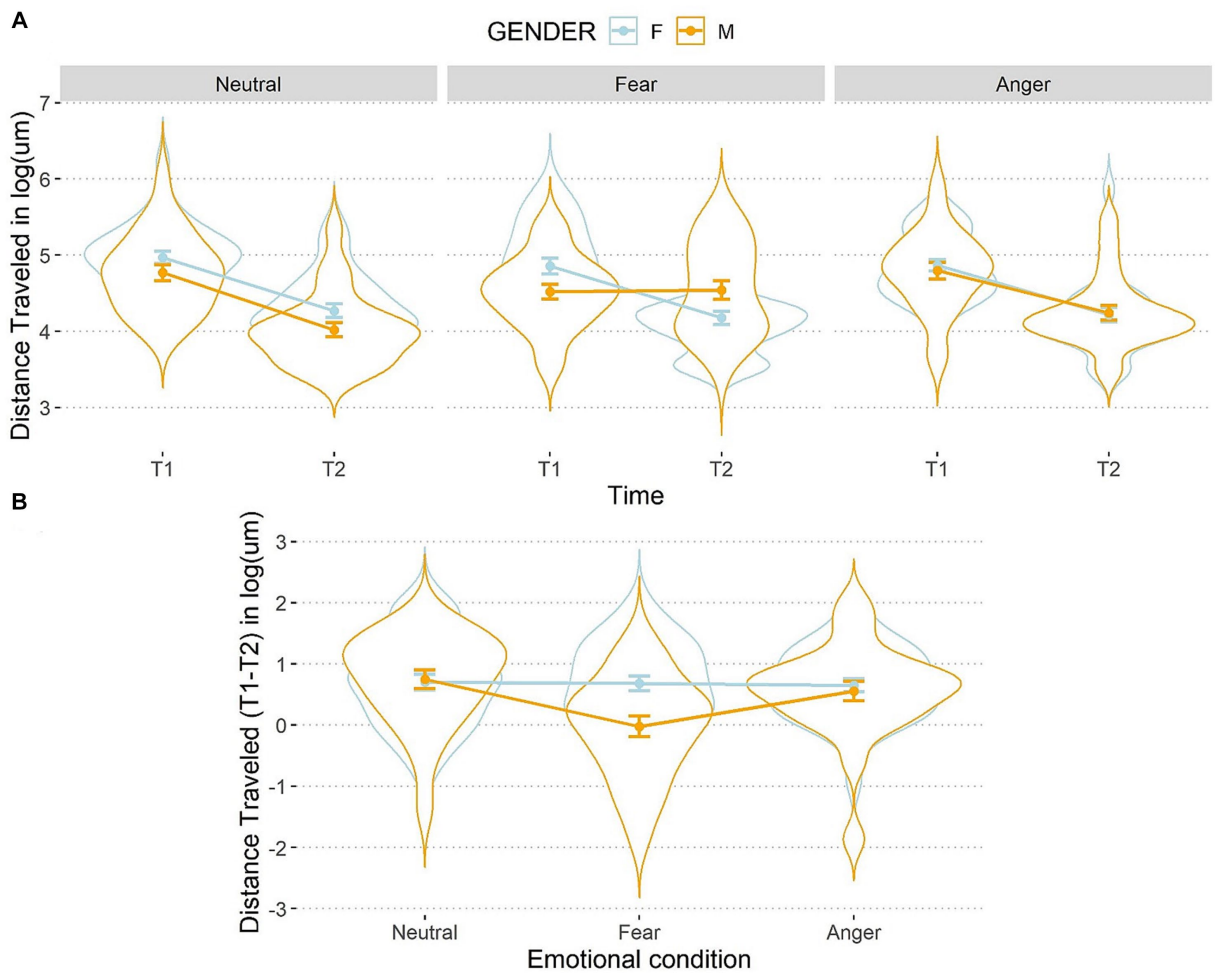
Line and violin plots representing the difference in distance travelled in seconds (after the logarithmic transformation) between single encoding (T1) and recalling (T2) sessions **(A)** and differential score (T1–T2) **(B)** as a function of gender and emotional conditions. Lower numbers indicate better performances in graph a, and vice versa in graph b. The light blue line represents female participants, and the orange line represents male participants. Bars represent standard error around the means.

Then, a significant interaction between emotions, time, and gender was found once again, *F*(2,106) = 4.32, *p* = 0.02, *η*^2^_p_ = 0.07 (see Figure 6).

A simple effects analysis on the three-way interaction showed that females and males improved their performances from T1 to T2 in all conditions, *t*(159) > 3.78, *p*s < 0.001, *d* > 0.30, but males did not show any difference between distance travelled at T1 and T2 after the fear manipulation, *t*(159) = −0.15, *p* = 0.88, *d* = 0.01. At T1, a simple effects analysis on females showed no differences between the three emotional conditions, *t*(211) < |0.91|, *p*s > 0.99, *d* < 0.06, as well as for males, *t*(211) < |1.96|, *p*s > 0.15, *d* < 0.13, showing that baseline performances in distance travelled were balanced between emotional conditions. At T2, females did not perform differently following the emotion manipulations, *t*(211) < |0.76|, *p*s > 0.99, *d* < 0.05. Conversely, at T2 a significant difference between the fearful and the neutral conditions was observed for males, *t*(211) = 3.72, *p* < 0.001, *d* = 0.26. This indicates that males travelled longer distances after the fearful condition. However, the difference was not markedly different between the fearful and the angry conditions, *t*(211) = −2.15, *p* = 0.06, *d* = 0.15, as well as the angry and the neutral conditions, *t*(211) = 1.57, *p* = 0.12, *d* = 0.11.

When looking at the differential scores (T1–T2), the results showed that, for females, distance travelled did not differ between the three emotional conditions, *t*(106) < −0.27, *p*s >0.99, *d* < 0.03, while males travelled longer distances at T2 than T1 after being exposed to fearful compared to angry, *t*(106) = 2.83, *p* = 0.01, *d* = 0.28, and to neutral faces, *t*(106) = −3.78, *p* < 0.001, *d* = 0.37. No difference emerged between the anger and neutral condition for males, *t*(106) = −0.95, *p* = 0.34, *d* = 0.09. Moreover, males travelled longer distances than females in the fearful condition, *t*(159) = 3.59, *p* < 0.001, *d* = 0.57, but no difference was observed when they were exposed to anger or neutral emotions, *t*(159) < 0.49, *p*s > 0.63, *d* < 0.08.

## Discussion

Despite emotions significantly impacting human behavior processes, the potential influence of emotion processing on spatial navigation has been largely overlooked. Hence, in the present research, we investigated how exposure to negative facial expressions affects wayfinding behaviors. In our study, participants navigated twice in three virtual reality environments (different floors of an office building). Each condition involved an initial encoding phase (i.e., autonomously learning a path to a target object) and a subsequent recalling phase (i.e., finding the same object placed in the same position of the previously explored environment). Between the two phases, faces showing one of the three emotional facial expressions (anger, fear, neutral) were presented for an unrelated task (i.e., gender categorization). Therefore, we measured travel times and distance travelled in the two phases as proxies for wayfinding behavioral performances.

Consistent with our hypothesis, participants were faster and travelled shorter distances in the recalling phase than in the encoding phase. Males were faster and travelled shorter distances than females on average, which might be in line with their higher wayfinding abilities (Coluccia and Louse, 2004). In fact, in the neutral condition – that is without any manipulation of emotion – males’ performances were better than females. The hypothesized effect of negative emotions on all participant’s navigation performance was observed, but it was better explained by a higher-order interaction with gender. Indeed, fearful faces disrupted only males’ wayfinding, while it was not true for females who were not affected. Angry and neutral faces had no effect on participants’ performances.

We observed that fearful and angry faces differed in their effect on the performance of males (but not females). A potential explanation relies in evolutionary-based behavioral reactions triggered by such emotions (e.g., approach/avoidance, fight/flight; Adams et al., 2006; Kreibig et al., 2007; Stins et al., 2011). For promoting survival, humans may be evolutionarily predisposed to prioritize the processing of threatening stimuli (Öhman et al., 2001; Rotteveel et al., 2015). In the broad context of navigation, perceiving emotional faces can be relevant for survival because they might be processed as social information for the surrounding environment (Hareli and Parkinson, 2008; Elfenbein, 2014). Using this framework of interpretation, participants exposed to other people expressing fear may have prioritized the detection of an unidentified threat within the surrounding context, either a person or an object (e.g., fire in the office). Even after the faces were no longer visible, the lingering sense of threat may have continued to impact their ability to navigate the surroundings. In contrast, anger often represents a threat that is more immediate and directed toward the observer in the present context. Indeed, angry faces may have been interpreted as indicating interpersonal threats (i.e., aggressive intentions), and once the faces disappeared, the perceived threat vanished and did not influence the subsequent navigation. This distinction suggests that the nature of the threat, whether directed at the observer or emanating from an unknown source, might play a crucial role in shaping an individual’s navigational responses. Future studies can address whether our speculations might justify our results.

Another possibility is that fearful facial expressions may have triggered emotion contagion, a process in which individuals tend to “catch” and experience similar negative emotions themselves. For example, observing another person’s fearful expression can lead to feelings of anxiety (Hatfield et al., 1993; Barsade, 2002). One of the consequences of emotion contagion may be imitation, which changes not only the individual’s emotional experience but also behavioral responses (Elfenbein, 2014). That is, participants exposed to fearful faces may have begun to feel a similar state (i.e., fear or anxiety) themselves, and such a feeling may have affected their wayfinding performance (for a review of emotion contagion, see Hatfield et al., 2014). Research on the effects of threat-induced anxiety has found that increased anxiety is associated with a decreased tendency to explore unfamiliar environments (Kallai et al., 2007; Newcombe, 2019; Bublatzky et al., 2023). But why were only males affected? One reason might be that females were better able to cope with emotion contagion than males, due to their more effective use of emotion regulation strategies (Goubet and Chrysikou, 2019). Indeed, the use of effective emotion regulation might have counteracted emotion contagion (Nilsonne et al., 2021). Therefore, new experimental evidence is needed to understand whether emotion contagion through repetitive exposure to fearful faces can indeed be a plausible explanation of our findings on wayfinding. For example, future research could measure participant’s sense of fear and anxiety induced by the emotional exposure to test whether these predict wayfinding behavior.

We might also speculate that negative emotion processing could have affected spatial navigation (in males) since the working memory (WM), and specifically the visuospatial working memory (VSWM), contribute both to facial emotion processing and spatial navigation (Dehn, 2011; Baddeley, 2012; Dickerson and Atri, 2014; Brown and Chrastil, 2019). The VSWM is responsible for processing and maintaining visuo-spatial information and plays a significant role in spatial navigation tasks, encompassing the identification of objects and their respective spatial locations (Garden et al., 2002; Coluccia and Louse, 2004; Nori et al., 2009). Additionally, processing negative emotions also demands considerable VSWM resources (Tyng et al., 2017). Specifically, fearful expressions have been associated with a detrimental effect on VSWM performances (see also Lindström and Bohlin, 2012; Berggren et al., 2017; Shields et al., 2017; Curby et al., 2019). Experiencing negative emotions can inhibit VSWM retention phases, affecting consolidation, and retrieval of spatial information (Shackman et al., 2006; Moran, 2016). For these reasons, it might be plausible to expect that the processing of negative facial expressions disrupted wayfinding by stealing people’s limited VSWM resources.

Moreover, although females usually underperform in wayfinding task (Coluccia and Louse, 2004), they might excel in emotional processing (Olderbak et al., 2019). Such superior ability in face emotion processing might have helped them in controlling the influence of fear on their wayfinding abilities. In addition, as exemplified above, females tend to use a larger variety of emotion regulation strategies than males (Goubet and Chrysikou, 2019). In contrast, males experienced a significant decline in performance after being exposed to fearful faces. To continue with the VSWM proposal, this could be due to the higher cognitive load on VSWM that fear processing imposed on men, whereas women found it less demanding and managed better the request. Future studies should investigate gender differences in VSWM performances in tasks involving emotional faces to address this open issue. Such results can also be investigated from a neurological perspective since gender dissimilarities in spatial and emotional processing are found also in brain lateralization of fundamental functions (González-Garrido et al., 2015; Yuan et al., 2019). In males, research found overlapping neural territories in the right hemisphere for emotion processing and spatial navigation, which may have led to higher resource competition during wayfinding (Castillo et al., 2021). In females, instead, there might have been an advantage due to the bilateral hemispheric activation reported for face processing, minimizing the aforementioned conflict (Proverbio et al., 2006). Being only speculation, future research should investigate our findings also from such perspectives.

Our research is not free from limitations. For instance, in our study, we exposed participants to emotional facial expressions without prompting them to actively assess the emotional content. This experimental design was chosen to investigate the influence of emotions on navigation when individuals are not asked by experimenters to engage in emotional processing, akin to scenarios where people traverse public spaces and encounter individuals displaying various facial expressions. Although individuals may not engage in explicit interpretation of these expressions, we posited that emotions could still exert an influence on the subsequent task through their repetitive exposure. Our approach might appear to run counter to the theoretical framework of appraisal theory (Moors and Fischer, 2019), which posits that emotional stimuli primarily impact human behavior when they are task-relevant, rather than when they are task-irrelevant. A comparison of task relevance was outside the scope of the present research; indeed, we did not include any task-relevant condition. However, our study might be compared to that of other researchers who investigated such issue with performance-based measures. Mirabella (2018), in a go no-go task centered on reaching arm movements, found that fearful and happy faces (compared to neutral faces) influenced behavior only when they were task relevant. Similar results were subsequently corroborated by Mancini et al. (2020) using also angry expressions (see also Mancini et al., 2022), and by Mirabella et al. (2023) in the context of whole-body movements (i.e., initiation of forward gait). However, there were notable disparities between our experimental approach and those employed by these studies. First, our task structure substantially differed from theirs, as we involved a repeated exposure to several emotional stimuli during the categorization task before measuring our primary outcome measures (i.e., times and distances travelled). In contrast, in their experiments, the measurement of emotional stimuli’s effect on behavior occurred simultaneously with the emotional stimulation. Second, our emotional conditions were independently presented, meaning that navigation occurred after exposure to each emotion individually. In contrast, the go no-go task presented facial emotions concurrently to examine their impact on behavior. Given these fundamental differences, a direct comparison of our results with those of the above-mentioned studies is challenging. Future research should evaluate the boundaries of the appraisal theory of emotions also for navigation behaviors.

We also acknowledge that our sample size was small and allowed limited inferences: future research should increase it to test with additional power our first investigation’s results. Moreover, in our research we did not consider the effects of positive emotions. Positive emotions convey different information and effects compared to negative ones, including stress reduction and recovery effects (Fredrickson and Branigan, 2005). They may reduce the intensity of behavioral responses and competition for the same cognitive resources, thereby enhancing and improving orienting abilities. In adjunct, we employed static pictures for manipulating emotions. This is less realistic than seeing someone’s emotional expression in the real world, which is often associated with the person’s body and its expressiveness. Future studies in VR could use digital avatars reproducing more realistic face-to-face interactions.

## Conclusion

Our results suggest that negative emotions can indeed influence spatial navigation. Fearful facial expressions impacted the wayfinding performance of males more significantly than both angry and neutral expressions, without a comparable effect on females. This gender-based distinction highlights the intricate interplay among emotion processing and navigational performances which might be due to several reasons. Based on these, we proposed several potential future investigations. Additionally, our study underscores the potential of virtual reality as a tool for investigating wayfinding abilities.

## Data availability statement

The datasets presented in this study can be found in online repositories. The names of the repository/repositories and accession number(s) can be found at: https://osf.io/wzbvy/.

## Ethics statement

The study was approved by the Committee for Research Evaluation of the Psychology Department of the University Milano-Bicocca (RM-2020-366). The participants provided their written informed consent to participate in this study.

## Author contributions

LM: Conceptualization, Data curation, Formal analysis, Investigation, Methodology, Project administration, Resources, Software, Visualization, Writing – original draft. MMa: Conceptualization, Formal analysis, Methodology, Writing – review & editing. MMo: Data curation, Software, Writing – review & editing. PR: Supervision, Writing – review & editing.

## Footnotes

^1^We also ran an identical pilot study (*N* = 20, see Supplementary material).
