# Supplementary material for: The effect of negative emotion processing on spatial navigation: an experimental study using virtual reality
Source: Front Psychol. 2024 Jan 11;14:1301981. doi: 10.3389/fpsyg.2023.1301981 (PMC10808736; doi:10.3389/fpsyg.2023.1301981)
Supplement: Supplementary file 1 [file Data_Sheet_1.PDF]

Supplementary Materials

The Effect of Negative Emotion Processing on Spatial Navigation:

An Experimental Study Using Virtual Reality

***Correspondence:** Corresponding Author l.mohamedaly@campus.unimib.it

Contents

Supplementary Materials 1: Pilot Study 3

Supplementary Materials 2: Assumptions Check of the Pilot Study 7

Supplementary Materials 3: Assumptions Check of the Main Study 15

Supplementary Materials 4: Exploratory Variables.. 24

Supplementary Materials 1: Pilot Study

We ran a pilot study with an identical design and procedure to that of the main study (see Methods section of the main text). We also collected the same exploratory variables of the main study at the end of the experimental session (see Supplementary Materials section about the exploratory variables) except for one variable (see SM1.3). Data are available on OSF.

Participants

Twenty-three students were recruited as participants. Three female subjects were excluded due to cybersickness. The final sample consisted of 20 participants (9 males, M_{age} = 27.3, SD_{age} = 3.81; 11 females, M_{age} = 26.8, SD_{age} = 3.60).

Data Preparation and statistical Analyses

As for the main study, assumptions for parametric tests were not respected for both travel times and distance travelled, therefore we applied a logarithmic transformation of the data (see Table SM1.1 and Table SM1.2 for data before and after the transformation). We then ran a 3 (emotion: neutral vs. fearful vs. angry faces) x 2 (time: encoding vs. recalling) x 2 (gender: female vs. male) mixed ANOVA on the two dependent variables.

Results

Results are reported in Table SM1.3. For both dependent variables, the only significant effect is that of time, showing that participants improved their performances the second time they entered the virtual environment. Although the three-way interaction between emotions, time, and gender was not significant, we can interpret the descriptive pattern. The emotion manipulations, both anger and fear, slightly reduced the performances of male participants with respect to the neutral condition and slightly increased that of females. Comparing the two genders, we observe that the performance of male participants decreases after the two emotional manipulations with respect to females, but it is higher in the neutral condition.

Because these were preliminary data and collected on a limited sample, we conducted the main study in which we doubled the sample size.

Table SM1.1 Means and standard deviation (in parenthesis) of travel time in seconds and travelled distances expressed in unit measures of the interaction between gender and the three emotional manipulations for the pilot study.

**Times**	*Female*			*Male*		
Emotion	T1	T2	T1-T2	T1	T2	T1-T2
Neutral	104 (54.0)	60.9 (22.0)	43.2 (56.1)	147 (103)	53.4 (26.1)	93.9 (112)
Fear	123 (67.5)	49.5 (18.1)	73.5 (62.8)	60.0 (19.5)	48.1 (38.5)	11.9 (46.7)
Anger	112 (52.2)	60.8 (55.1)	61.1 (49.8)	67.1 (36.2)	44.6 (23.9)	22.6 (44.0)
**Distances**	*Female*			*Male*		
Emotion	T1	T2	T1-T2	T1	T2	T1-T2
Neutral	96.6 (40.8)	80.6 (29.8)	16.0 (37.7)	214 (123)	99.1 (49.5)	114.9 (163)
Fear	115 (58.3)	63.6 (17.2)	51.0 (64.8)	95.8 (28.9)	94.9 (94.7)	0.90 (102.0)
Anger	127 (69.5)	79.7 (74.4)	46.8 (72.8)	105 (58.5)	84.4 (50.2)	21.1 (79.5)

Table SM1.2 Means and standard deviation (in parenthesis) of travel time in seconds and travelled distances expressed in unit measures of the interaction between gender and the three emotional manipulations for the pilot study after the logarithmic transformation.

**Times (log)**	*Female*			*Male*		
Emotion	T1	T2	T1-T2	T1	T2	T1-T2
Neutral	4.51 (.56)	4.05 (.34)	.45 (.57)	4.76 (.73)	3.82 (.66)	.94 (1.10)
Fear	4.67 (.57)	3.83 (.41)	.84 (.58)	4.05 (.30)	3.66 (.65)	.38 (.76)
Anger	4.71 (.46)	3.86 (.67)	.84 (.50)	4.10 (.48)	3.67 (.56)	.42 (.75)
**Distances (log)**	*Female*			*Male*		
Emotion	T1	T2	T1-T2	T1	T2	T1-T2
Neutral	4.50 (.39)	4.34 (.33)	.16 (.40)	5.21 (.60)	4.46 (.58)	.75 (1.12)
Fear	4.63 (.49)	4.12 (.27)	.51 (.59)	4.52 (.31)	4.30 (.67)	.22 (.76)
Anger	4.71 (.53)	4.17 (.59)	.54 (.71)	4.56 (.44)	4.30 (.53)	.26 (.71)

Table SM1.3. Results of the Statistical Analysis on Behavioral outcomes of the Pilot Study.

Variable	Effect	Factors	*df*	*F*	*p*	ES
Time	Main	Time	1, 18	44.6	**.001**	.71
	Main	Gender	1, 18	3.73	.06	.17
	Main	Emotion	2, 36	2.69	.08	.13
	Interaction	Time x Gender	1, 18	.42	.52	.02
	Interaction	Time x Emotion	2, 36	.08	.92	.005
	Interaction	Emotion x Gender	2, 36	2.32	.11	.11
	Interaction	Time x Gender x Emotion	2, 36	2.81	.07	.13
Distances	Main	Time	1, 18	17.7	**.001**	.50
	Main	Gender	1, 18	2.89	.10	.14
	Main	Emotion	2, 36	2.61	.08	.13
	Interaction	Time x Gender	1, 18	.001	.97	.000
	Interaction	Time x Emotion	2, 36	.08	.92	.005
	Interaction	Emotion x Gender	2, 36	2.27	.12	.11
	Interaction	Time x Gender x Emotion	2, 36	2.70	.08	.13

Supplementary Materials 2: Assumptions Check of the Pilot Study

We checked whether the assumptions of parametric tests (i.e., mixed ANOVA) were respected in our data. We report here the results. Since for both our dependent variables, travel times and distance travelled, they were not respected (see table and figure SM2.1, SM2.3), we proceeded to transform the data to normalize the distributions. We then checked again the assumptions and they were respected (see table and figure SM2.2, SM2.4).

Note that in the tables' variables names: N stands for Neutral, F for Fear, and A for Anger, 1 for T1, and 2 for T2, unless otherwise stated.

SM2.1 Assumptions Travel Time Pre-Transformation

Tests of Sphericity.

	Mauchly's W	*p*	*Greenhouse-Geisser ε*	*Huynh-Feldt ε*
Emotion	0.974	0.802	0.975	1.000
Time	1.000	NaN [a]	1.000	1.000
Emotion ✻ Time	0.815	0.176	0.844	0.922

[a] The repeated measures has only two levels. The assumption of sphericity is always met when the repeated measures has only two levels.

Homogeneity of Variances Test (Levene's).

	F	*df1*	*df2*	*p*
N1	6.219	1	18	0.023
N2	0.758	1	18	0.395
F1	7.049	1	18	0.016
F2	1.622	1	18	0.219
A1	2.813	1	18	0.111
A2	1.474	1	18	0.240

Shapiro Wilk test on the cells of 2x2x3 Mixed ANOVA checking the normality of the distributions.

	Gender	*N1*	*N2*	*F1*	*F2*	*A1*	*A2*
W	F	0.929	0.801	0.934	0.976	0.926	0.675
	M	0.854	0.736	0.889	0.738	0.799	0.907
p	F	0.398	0.010	0.447	0.942	0.370	< .001
	M	0.083	0.004	0.193	0.004	0.020	0.293

Q-Q Plot on the residuals of the 2x2x3 Mixed ANOVA for the normality of residuals.

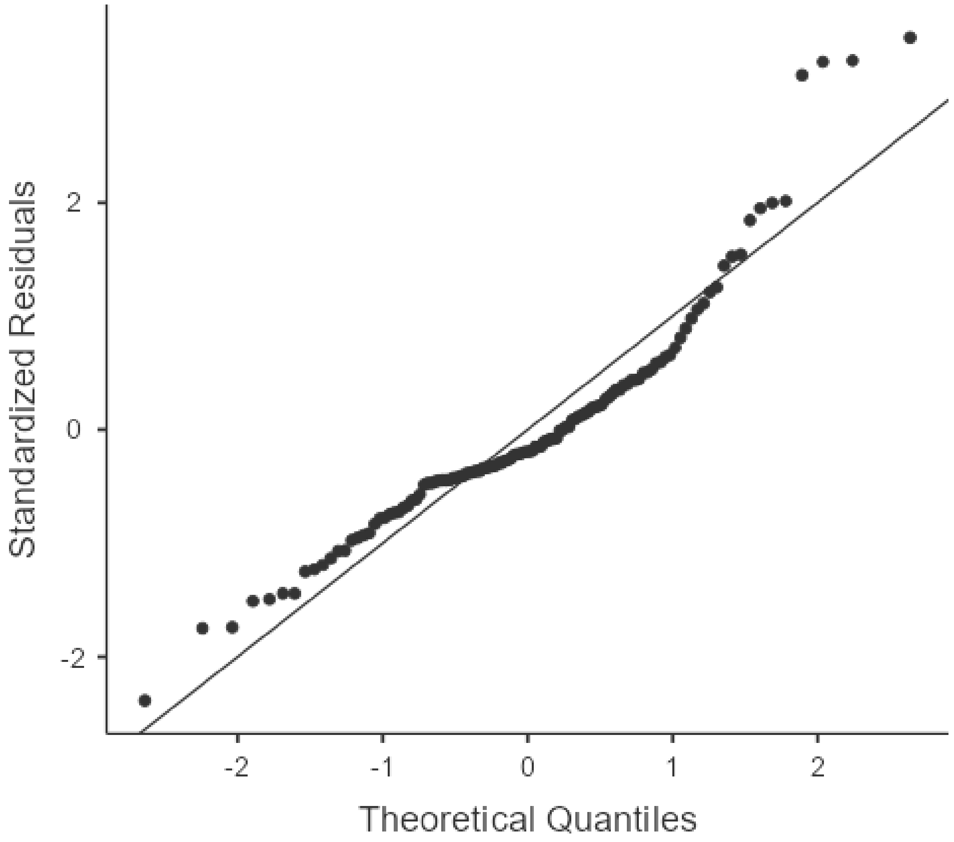

SM2.2 Assumptions Travel Time Post Log Transformation

Tests of Sphericity.

	Mauchly's W	*p*	*Greenhouse-Geisser ε*	*Huynh-Feldt ε*
Emotions	0.976	0.814	0.977	1.000
Time	1.000	NaN [a]	1.000	1.000
Emotions ✻ Time	0.769	0.107	0.812	0.881

[a] The repeated measures has only two levels. The assumption of sphericity is always met when the repeated measures has only two levels.

Homogeneity of Variances Test (Levene's).

	F	*df1*	*df2*	*p*
N1 - Log	0.7655	1	18	0.393
N2 - Log	11.7869	1	18	0.003
F1 - Log	4.3429	1	18	0.052
F2 - Log	1.1135	1	18	0.305
A1 - Log	0.1214	1	18	0.732
A2 - Log	0.0887	1	18	0.769

Shapiro Wilk test on the cells of 2x2x3 Mixed ANOVA checking the normality of the distributions.

	**Gender**	*N1*	*N2*	*F1*	*F2*	*A1*	*A2*
W	F	0.958	0.827	0.979	0.946	0.954	0.934
	M	0.946	0.708	0.936	0.947	0.905	0.961
p	F	0.744	0.021	0.959	0.598	0.698	0.457
	M	0.644	0.002	0.544	0.654	0.282	0.810

Q-Q Plot on the residuals of the 2x2x3 Mixed ANOVA for the normality of residuals.

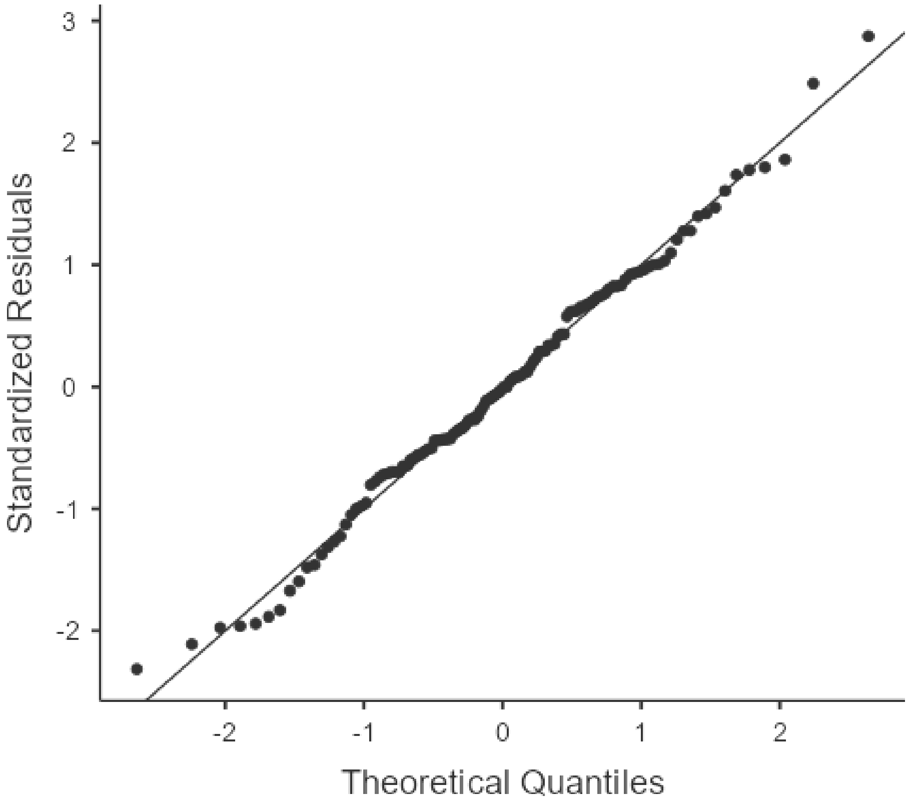

SM2.3 Assumptions Distance Travelled Pre-Transformation

Tests of Sphericity.

	Mauchly's W	*p*	*Greenhouse-Geisser ε*	*Huynh-Feldt ε*
Emotions	0.970	0.773	0.971	1.000
Time	1.000	NaN [a]	1.000	1.000
Emotions ✻ Time	0.765	0.102	0.809	0.878

[a] The repeated measures has only two levels. The assumption of sphericity is always met when the repeated measures has only two levels.

Homogeneity of Variances Test (Levene's).

	F	*df1*	*df2*	*p*
N1	3.6486	1	18	0.072
N2	8.2558	1	18	0.010
F1	4.1328	1	18	0.057
F2	4.0050	1	18	0.061
A1	1.2409	1	18	0.280
A2	0.0863	1	18	0.772

Shapiro Wilk test on the cells of 2x2x3 Mixed ANOVA checking the normality of the distributions.

	Gender	*N1*	*N2*	*F1*	*F2*	*A1*	*A2*
W	F	0.833	0.830	0.894	0.960	0.893	0.556
	M	0.900	0.857	0.950	0.598	0.638	0.839
p	F	0.026	0.023	0.155	0.774	0.152	< .001
	M	0.255	0.089	0.688	< .001	< .001	0.057

Q-Q Plot on the residuals of the 2x2x3 Mixed ANOVA for the normality of residuals.

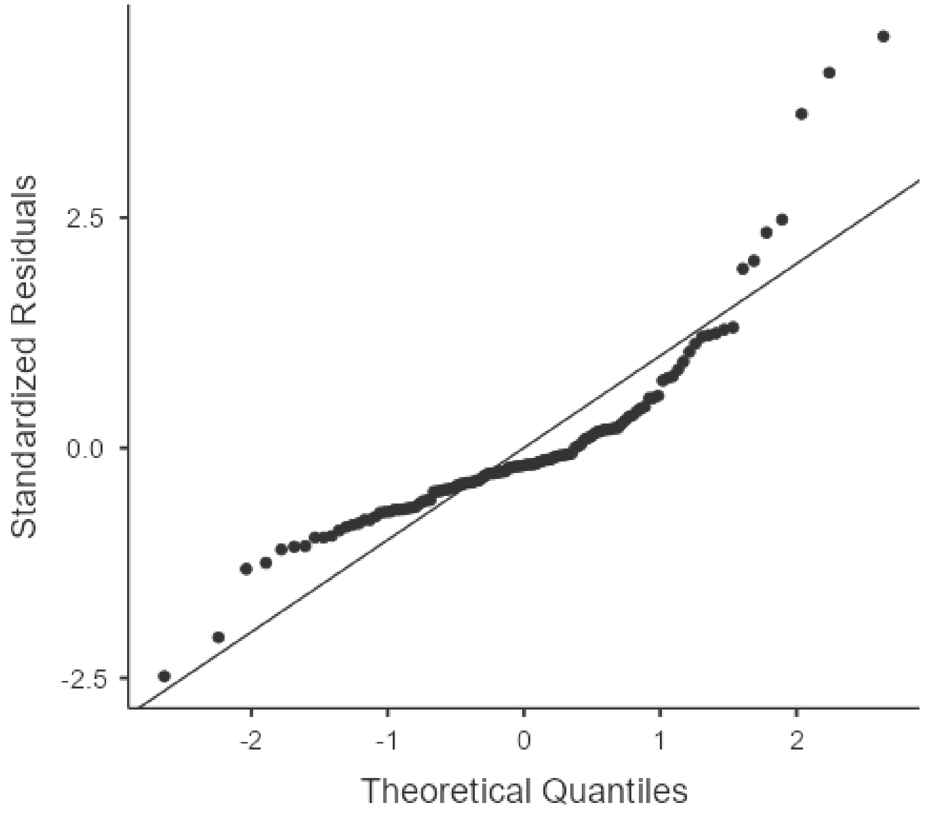

SM2.4 Assumptions Distance Travelled Post-Transformation

Tests of Sphericity.

	Mauchly's W	*p*	*Greenhouse-Geisser ε*	*Huynh-Feldt ε*
Emotions	0.809	0.165	0.840	0.916
Time	1.000	NaN [a]	1.000	1.000
Emotions ✻ Time	0.741	0.078	0.794	0.859

[a] The repeated measures has only two levels. The assumption of sphericity is always met when the repeated measures has only two levels.

Homogeneity of Variances Test (Levene's).

	F	*df1*	*df2*	*p*
N1 - Log	1.4891	1	18	0.238
N2 - Log	9.7030	1	18	0.006
F1 - Log	2.6105	1	18	0.124
F2 - Log	2.5588	1	18	0.127
A1 - Log	1.3399	1	18	0.262
A2 - Log	0.0134	1	18	0.909

Shapiro Wilk test on the cells of 2x2x3 Mixed ANOVA checking the normality of the distributions.

	Gender	*N1*	*N2*	*F1*	*F2*	*A1*	*A2*
W	F	0.911	0.911	0.935	0.970	0.966	0.824
	M	0.956	0.860	0.961	0.853	0.799	0.984
p	F	0.252	0.253	0.468	0.882	0.848	0.020
	M	0.756	0.096	0.807	0.080	0.020	0.981

Q-Q Plot on the residuals of the 2x2x3 Mixed ANOVA for the normality of residuals.

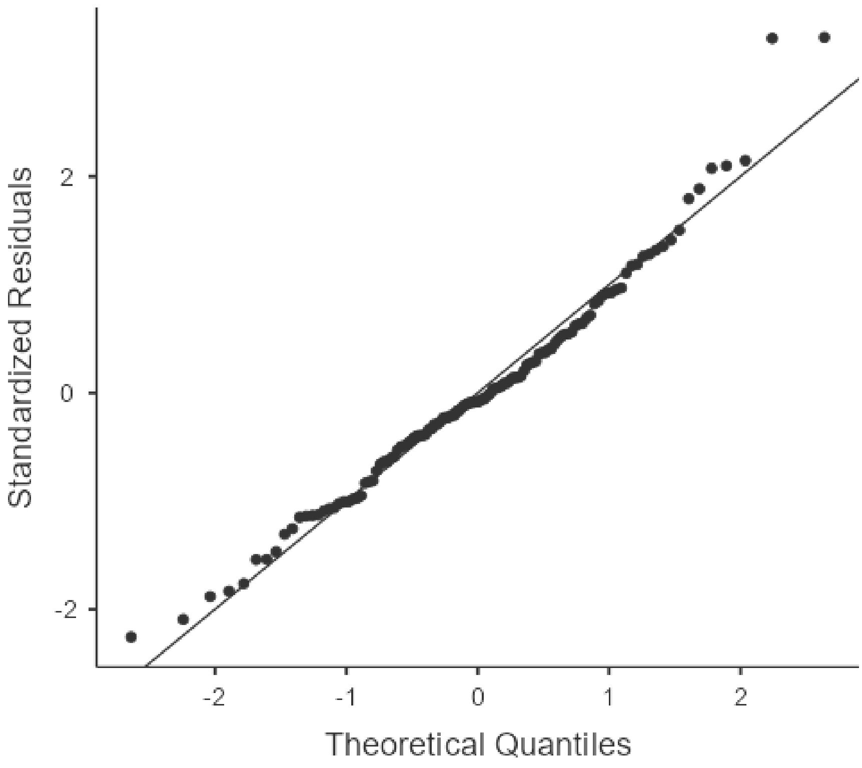

Supplementary Materials 3: Assumptions Check of the Main Study

We checked whether the assumptions of parametric tests (i.e., mixed ANOVA) were respected in our data. We report here the results. Since for both our dependent variables, travel times and distance travelled, they were not respected (see table and figure SM3.1, SM3.3), we proceeded to transform the data to normalize the distributions. We then checked again the assumptions and they were respected (see table and figure SM3.2, SM3.4).

Note that in the tables' variables names: N stands for Neutral, F for Fear, and A for Anger, 1 for T1, and 2 for T2, unless otherwise stated.

SM3.1 Assumptions Travel Time Pre-Transformation

Tests of Sphericity.

	Mauchly's W	*p*	*Greenhouse-Geisser ε*	*Huynh-Feldt ε*
Emotions	0.893	0.052	0.903	0.933
Time	1.000	NaN [a]	1.000	1.000
Emotions ✻ Time	0.891	0.050	0.902	0.932

[a] The repeated measures has only two levels. The assumption of sphericity is always met when the repeated measures has only two levels.

Homogeneity of Variances Test (Levene's).

	F	*df1*	*df2*	*p*
N1	0.216	1	53	0.644
N2	2.585	1	53	0.114
F1	10.356	1	53	0.002
F2	0.242	1	53	0.625
A1	3.075	1	53	0.085
A2	6.632	1	53	0.013

Shapiro Wilk test on the cells of 2x2x3 Mixed ANOVA checking the normality of the distributions.

	Gender	*N1*	*N2*	*F1*	*F2*	*A1*	*A2*
W	F	0.648	0.860	0.804	0.658	0.906	0.641
	M	0.795	0.741	0.941	0.866	0.819	0.862
p	F	< .001	< .001	< .001	< .001	0.010	< .001
	M	< .001	< .001	0.168	0.004	< .001	0.004

Q-Q Plot on the residuals of the 2x2x3 Mixed ANOVA for the normality of residuals.

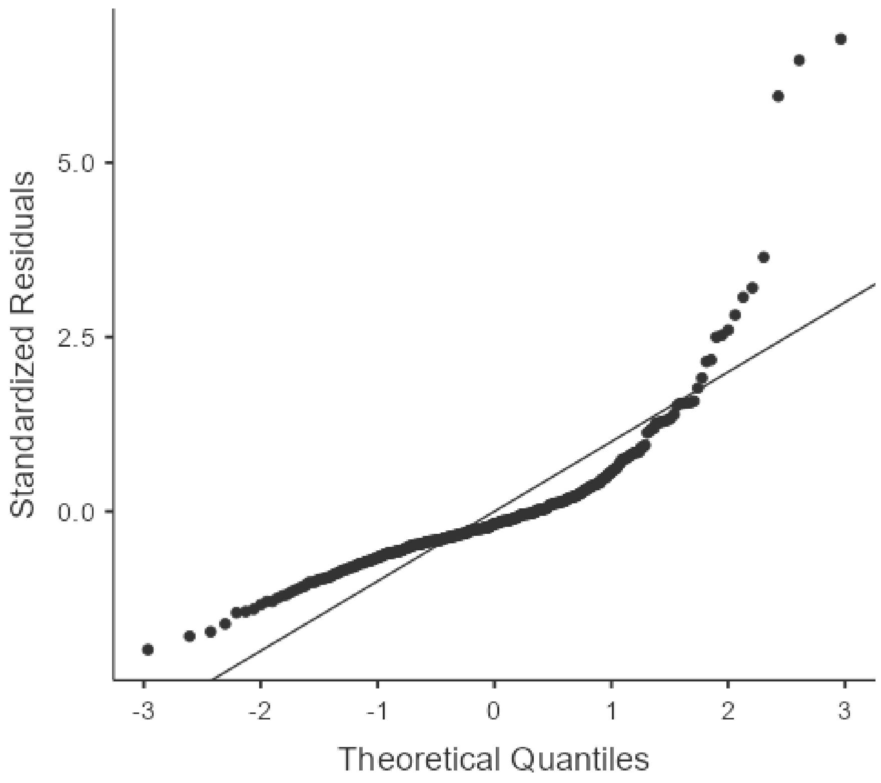

SM3.2 Assumptions Time Travel Post Transformation

Tests of Sphericity

	Mauchly's W	*p*	*Greenhouse-Geisser ε*	*Huynh-Feldt ε*
Emotions	1.000	0.992	1.000	1.000
Time	1.000	NaN [a]	1.000	1.000
Emotions ✻ Time	0.912	0.092	0.919	0.951

[a] The repeated measures has only two levels. The assumption of sphericity is always met when the repeated measures has only two levels.

Homogeneity of Variances Test (Levene's)

	F	*df1*	*df2*	*p*
N1 - Log	1.141	1	53	0.290
N2 - Log	0.345	1	53	0.560
F1 - Log	0.280	1	53	0.599
F2 - Log	0.912	1	53	0.344
A1 - Log	0.507	1	53	0.480
A2 - Log	3.849	1	53	0.055

Shapiro Wilk test on the cells of 2x2x3 Mixed ANOVA checking the normality of the distributions

	Gender	*N1 - Log*	*N2 - Log*	*F1 - Log*	*F2 - Log*	*A1 - Log*	*A2 - Log*
W	F	0.931	0.985	0.988	0.944	0.973	0.917
	M	0.933	0.917	0.948	0.933	0.975	0.982
p	F	0.046	0.938	0.975	0.105	0.619	0.020
	M	0.113	0.049	0.246	0.114	0.779	0.935

Q-Q Plot on the residuals of the 2x2x3 Mixed ANOVA for the normality of residuals.

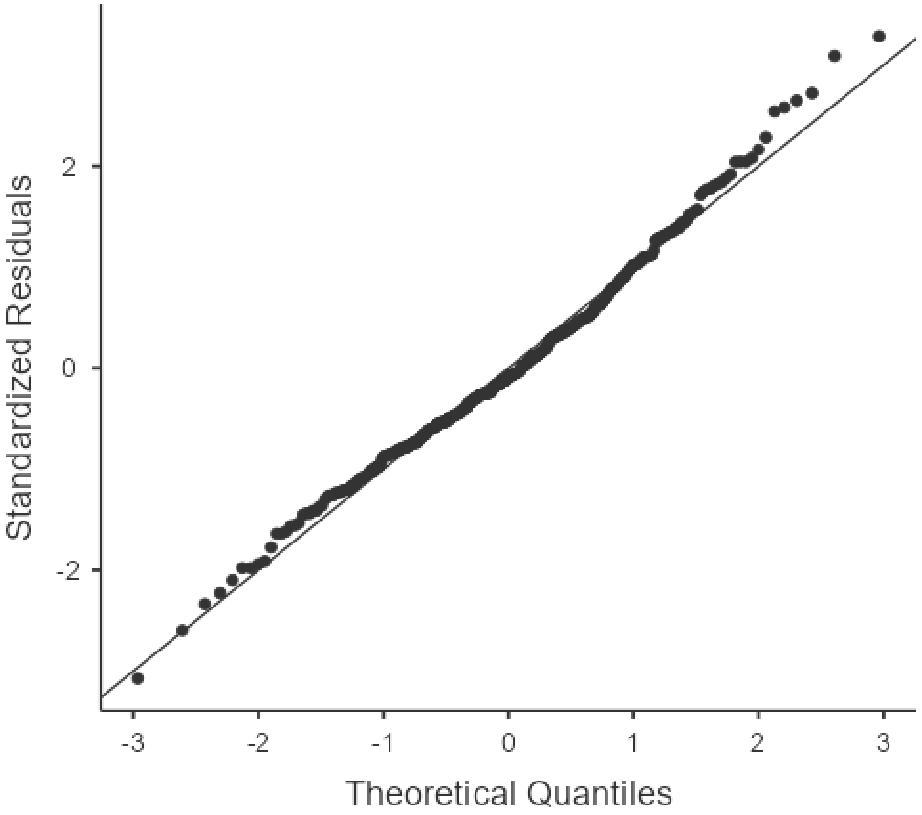

SM3.3 Assumptions Distance Travelled Pre-Transformation

Tests of Sphericity

	Mauchly's W	*p*	*Greenhouse-Geisser ε*	*Huynh-Feldt ε*
Emotions	0.993	0.836	0.993	1.000
Time	1.000	NaN [a]	1.000	1.000
Emotions ✻ Time	0.941	0.205	0.944	0.978

[a] The repeated measures has only two levels. The assumption of sphericity is always met when the repeated measures has only two levels.

Homogeneity of Variances Test (Levene's)

	F	*df1*	*df2*	*p*
N1-DIST	0.00170	1	53	0.967
N2-DIST	0.75549	1	53	0.389
F1-DIST	7.07865	1	53	0.010
F2-DIST	6.69291	1	53	0.012
A1-DIST	0.02119	1	53	0.885
A2-DIST	0.25861	1	53	0.613

Shapiro Wilk test on the cells of 2x2x3 Mixed ANOVA checking the normality of the distributions.

	Gender	*N1*	*N2*	*F1*	*F2*	*A1*	*A2*
W	F	0.776	0.816	0.923	0.706	0.885	0.615
	M	0.810	0.695	0.907	0.871	0.870	0.711
p	F	< .001	< .001	0.028	< .001	0.003	< .001
	M	< .001	< .001	0.031	0.006	0.005	< .001

Q-Q Plot on the residuals of the 2x2x3 Mixed ANOVA for the normality of residuals.

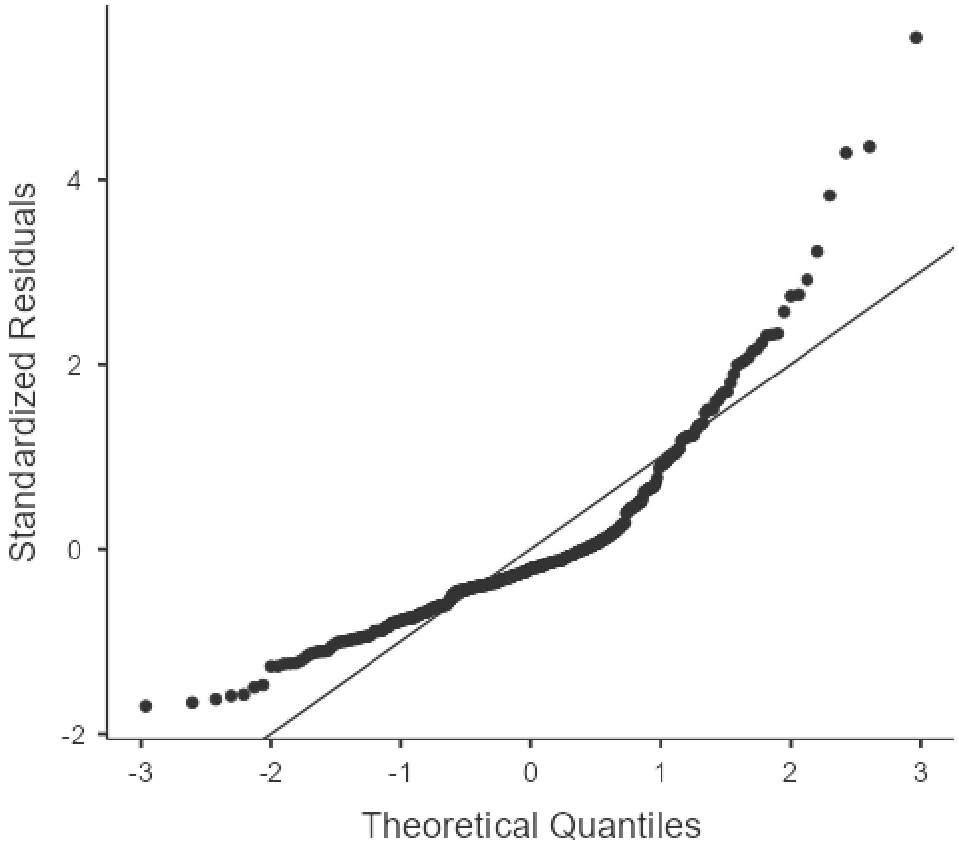

SM3.4 Assumptions Distance Travelled Post Transformation

Tests of Sphericity

	Mauchly's W	p	Greenhouse-Geisser ε	Huynh-Feldt ε
Emotions	0.974	0.508	0.975	1.000
Time	1.000	NaN [a]	1.000	1.000
Emotions ✻ Time	0.934	0.169	0.938	0.971

[a] The repeated measures has only two levels. The assumption of sphericity is always met when the repeated measures has only two levels.

Homogeneity of Variances Test (Levene's)

	F	df1	df2	p
N1-DIST - Log	0.394	1	53	0.533
N2-DIST - Log	0.185	1	53	0.669
F1-DIST - Log	1.572	1	53	0.215
F2-DIST - Log	3.741	1	53	0.058
A1-DIST - Log	0.322	1	53	0.573
A2-DIST - Log	0.284	1	53	0.597

Shapiro Wilk test on the cells of 2x2x3 Mixed ANOVA checking the normality of the distributions.

	Gender	N1-DIST - Log	N2-DIST - Log	F1-DIST - Log	F2-DIST - Log	A1-DIST - Log	A2-DIST - Log
W	F	0.971	0.962	0.968	0.924	0.940	0.885
	M	0.967	0.899	0.981	0.928	0.976	0.904
p	F	0.547	0.322	0.469	0.031	0.083	0.003
	M	0.597	0.021	0.913	0.089	0.813	0.026

Q-Q Plot on the residuals of the 2x2x3 Mixed ANOVA for the normality of residuals.

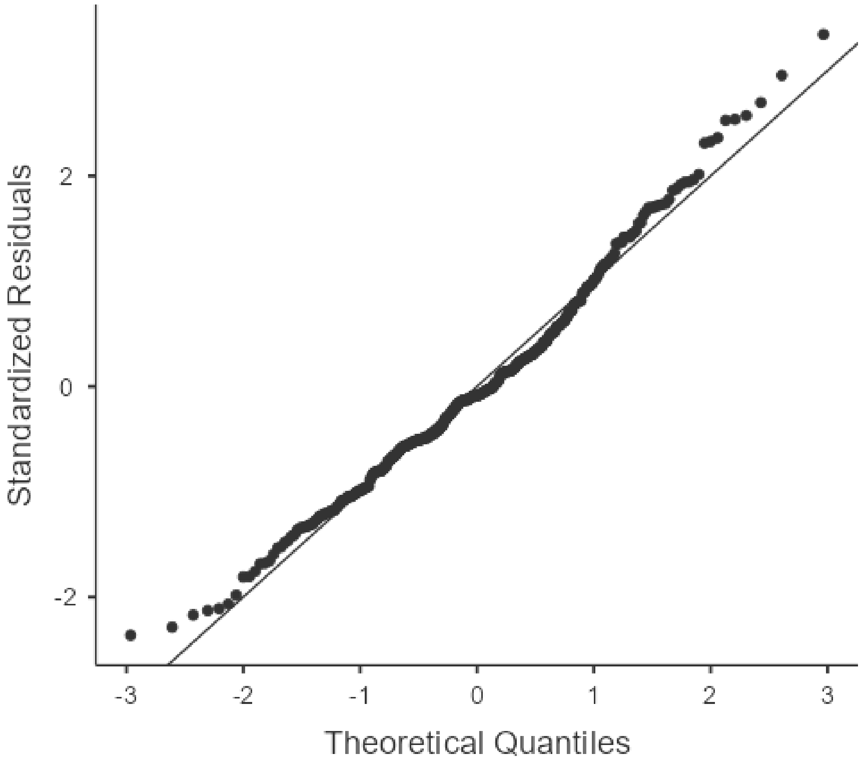

Supplementary Materials 4: Exploratory Variables

In both the pilot study and the main study, we focused on exploratory variables to explore the characteristics of our sample and to inspect potentially interesting relationships between navigation, emotions, and individual characteristic. The questionnaires were administered at the end of the experimental phase to not affect the outcome of our main results. We report here a description of the measures, the descriptive analyses results, and a partial correlation table separated for male and female participants.

Materials

Pilot and Main study

Simulator Sickness Questionnaire (SSQ; Walter et al., 2019). To evaluate cybersickness related to the VR tasks, participants rated the severity of each symptom (e.g., nausea, headache, sweating) on a 4-point Likert scale (0 "None" to 3 "Severe") using the 16 items of the The SSQ provided four representative sub-scores: nausea-related (N), oculomotor-related (O), disorientation-related (D), and a total score (TS).

Autism Spectrum Quotient scale (AQ; Baron-Cohen et al., 2005). We included this measure to explore the potential relationship of the presence of autistic traits with wayfinding behaviours and navigation after our emotional manipulations. In autism, navigation is atypical as well as emotion processing (Ring et al., 2018; Baron-Cohen et al., 2005) and individuals with high autistic traits may share the same characteristic. Therefore, it seemed plausible to us that participants with high autistic traits might also be less performant in navigation even before the emotional exposure. The AQ questionnaire consists of 50 items. The AQ can identify individuals who may not meet diagnostic criteria for autism, but who exhibit high autistic traits that may influence the effect of being exposed to emotional stimuli since, in autism, emotional processing is often atypical or reduced. All our participants were below the cut-off level set by the developers thought to indicate a higher risk and probability to meet the criteria for the diagnosis of autism.

Pilot study

Depression, Anxiety, Stress State scale (DASS-21; Samani & Joukar, 2007; Medvedev, 2023). We explored the relationship between self-reported emotional states and the results of our experiment. For measuring the emotional states of stress, anxiety, and depression. The short version of the DASS is a set of three self-report scales, based on a dimensional rather than a categorical conception of each psychological disorder. Each of the three scales contains 7 items, divided into subscales with similar content. The depression scale assessed dysphoria, hopelessness, devaluation of life, self- deprecation, lack of interest/involvement, anhedonia, and inertia. The anxiety scale assessed autonomic arousal, skeletal muscle effects, situational anxiety, and subjective experience of anxious affect. While the stress scale assessed difficulty relaxing, nervous arousal, and being easily upset/agitated, irritable/over-reactive and impatient.

Main study

Wayfinding Questionnaire (WQ; Claessen et al. 2016). The questionnaire was used to assess participants' self-reported navigation abilities. It contains 22 items divided into three subscales: navigation and orientation (11 items), distance estimation (3 items), and spatial anxiety (8 items). Refer to Claessen et al. 2016 for scoring rules.

Results

Pilot Study

The correlation table shows that for female participants there is no correlation between exploratory variables and both time travel and distances travelled. For male participants, there is a negative association between the subscale's "depression" and "anxiety" of the DASS-21 and the performance in the fear condition, such that higher scores in depression and anxiety were associated with lower performances in fear.

Main study

The correlation table shows that for female participants there is a positive association between simulation sickness (SSQ) and distance travelled in the fear condition, but not with any other conditions. For male participants, there is a negative association between the subscales "orientation and navigation" and "distance estimation" of the WQ and the performance in the fear condition, this means that higher scores in the variables were associated with a decrease in performance after the fear exposure. Also, there was a positive association between the subscale spatial anxiety and the performance in the neutral condition.

Discussion

These data were collected to explore the possible patterns of correlations between navigation, emotions, and psychological self-reported measures. Our results suggest that emotional and wayfinding self-reported characteristics of participants correlated with some of the results we obtained in males but not in female participants. Males are also the participants that were most affected by our emotion conditions (see Main Text for details). For this reason, future studies should systematically investigate the implications of these variables for navigation and emotion processing.

References

Baron-Cohen, S., Wheelwright, S., Skinner, R., Martin, J., & Clubley, E. (2001). The autism-spectrum quotient (AQ): Evidence from Asperger syndrome/high-functioning autism, males and females, scientists, and mathematicians. *Journal of Autism and Developmental disorders*, 31(1), pp. 5-17.

Claessen, M.H., Visser-Meily, J.M., de Rooij, N.K., Postma, A., & van der Ham, I.J. (2016). The wayfinding questionnaire as a self-report screening instrument for navigation-related complaints after stroke: Internal validity in healthy respondents and chronic mild stroke patients. *Archives of Clinical Neuropsychology*, 31(8), pp. 839-854.

Medvedev, O. N. (2023). Depression Anxiety Stress Scales (DASS-21) in International Contexts. In C. U. Krägeloh et al. (Eds.). *International Handbook of Behavioral Health Assessment* (pp. 1-15). Cham: Springer International Publishing.

Samani, S., & Joukar, B. (2007). A study on the reliability and validity of the short form of the depression anxiety stress scale (DASS-21).

Walter, H., Li, R., Munafo, J., Curry, C., Peterson, N., & Stoffregen, T. (2019). *APAL coupling study 2019. Simulator Sickness Questionnaire (SSQ).* Retrieved from the Data Repository for the University of Minnesota, https://doi.org/10.13020/XAMG-CS69.

Table SM4.1. Descriptive analysis of the exploratory variables of the pilot study

	Gender	Age	SSQ	AQ	DASS-21 Depression	DASS-21 Anxiety	DASS- 21 Stress
N	F	11	11	11	11	11	11
	M	9	9	9	9	9	9
Missing	F	0	0	0	0	0	0
	M	0	0	0	0	0	0
Mean	F	26.8	62.6	13.0	11.8	8.73	14.4
	M	27.3	39.1	16.2	10.0	6.67	18.9
Median	F	27	48.6	12	12	8	12
	M	26	26.2	16	8	4	18
Standard deviation	F	3.60	42.2	5.39	5.40	7.76	7.15
	M	3.81	34.9	4.02	6.56	8.60	7.88
Minimum	F	20	11.2	3	0	0	2
	M	24	3.74	8	2	0	4
Maximum	F	33	146	21	20	30	30
	M	37	112	22	24	24	30

Table SM4.2. Descriptive analysis of the exploratory variables of the main study.

	Gender	Age	SSQ	AQ	WQ - Navigation & Orientation	WQ – Spatial Anxiety	WQ – Distance Estimation
N	F	31	30	31	31	31	31
	M	24	24	24	24	24	24
Mean	F	21.5	40.9	16.1	50.5	34.2	9.90
	M	23.5	19.3	18.5	58.5	39.3	13.8
Median	F	22	37.4	16	49	34	10
	M	23.0	18.7	18.5	61.0	39.0	13.5
SD	F	2.55	24.2	5.98	10.3	9.91	4.35
	M	2.72	14.5	6.60	11.9	8.45	4.08
Minimum	F	18	3.74	4	27	17	3
	M	19	0.00	6	31	22	7
Maximum	F	28	97.2	30	72	56	20
	M	29	56.1	32	76	56	21

Table SM4.3. Female participants. Partial correlation table including the experimental dependent variables and the exploratory variables of the pilot study.

Correlation - Pearson's r

	AGE	T-N	T-F	T-A	D-N	D-F	D-A	DASS-1	DASS-2	DASS-3	AQ	SSQ
**AGE**	—											
**T-N**	-0.062	—										
**T-F**	-0.239	0.156	—									
**T-A**	-0.305	-0.582	-0.198	—								
**D-N**	0.054	0.953 ***	0.115	-0.645 *	—							
**D-F**	-0.266	-0.015	0.952 ***	-0.129	-0.001	—						
**D-A**	-0.432	-0.581	-0.118	0.896 ***	-0.581	0.026	—					
**DASS-1**	0.039	0.196	0.233	-0.023	0.209	0.189	-0.191	—				
**DASS-2**	-0.482	0.167	0.162	0.290	0.158	0.239	0.406	0.443	—			
**DASS-3**	-0.293	0.156	0.104	0.128	0.131	0.142	0.075	0.810 **	0.817 **	—		
**AQ**	-0.144	0.228	0.385	-0.248	0.178	0.262	-0.408	0.750 **	0.101	0.520	—	
**SSQ**	-0.151	0.051	-0.365	0.196	0.064	-0.402	0.151	0.321	-0.043	0.125	0.202	—

Note. * $p < .05$, ** $p < .01$, *** $p < .001$. T = travel times (T1-T2), D = distance travelled (T1-T2), N = neutral, F = fear, A = Anger, DASS-1 = Depression, DASS-2 = Anxiety, DASS-3 = Stress, AQ = Autism Spectrum Quotient scale, SSQ = Simulator Sickness Questionnaire.

Table SM4.4. Male participants. Partial correlation table including the experimental dependent variables and the exploratory variables of the pilot study.

Correlation - Pearson's r

	AGE	T-N	T-F	T-A	D-N	D-F	D-A	DASS-1	DASS-2	DASS-3	AQ	SSQ
**AGE**	—											
**T-N**	-0.294	—										
**T-F**	0.142	-0.074	—									
**T-A**	-0.191	-0.006	0.729 *	—								
**D-N**	-0.256	0.984 ***	-0.105	0.006	—							
**D-F**	0.017	-0.087	0.975 ***	0.710 *	-0.136	—						
**D-A**	-0.114	0.024	0.803 **	0.972 ***	0.052	0.781 *	—					
**DASS-1**	-0.370	-0.138	-0.798 **	-0.416	-0.093	-0.762 *	-0.508	—				
**DASS-2**	-0.321	-0.232	-0.766 *	-0.636	-0.210	-0.701 *	-0.689 *	0.913 ***	—			
**DASS-3**	-0.178	-0.343	-0.438	-0.379	-0.331	-0.460	-0.457	0.774 *	0.816 **	—		
**AQ**	-0.707 *	-0.082	-0.429	-0.299	-0.124	-0.349	-0.409	0.540	0.660	0.576	—	
**SSQ**	-0.033	0.100	0.176	0.084	0.174	0.033	0.123	-0.057	-0.017	0.289	0.253	—

Note. * p < .05, ** p < .01, *** p < .001. T = travel times (T1-T2), D = distance travelled (T1-T2), N = neutral, F = fear, A = Anger, DASS-1 = Depression, DASS-2 = Anxiety, DASS-3 = Stress, AQ = Autism Spectrum Quotient scale, SSQ = Simulator Sickness Questionnaire

Table SM4.5. Female participants. Partial correlation table including the experimental dependent variables and the exploratory variables of the main study.

Correlation - Pearson's r

	AGE	T-N	T-F	T-A	D-N	D-F	D-A	WQ1	WQ2	WQ3	AQ	SSQ
**AGE**	—											
**T-N**	0.226	—										
**T-F**	0.004	-0.069	—									
**T-A**	-0.103	0.178	-0.546 **	—								
**D-N**	0.128	0.839 ***	-0.036	0.113	—							
**D-F**	0.028	-0.111	0.901 ***	-0.504 **	-0.102	—						
**D-A**	0.042	0.143	-0.526 **	0.804 ***	0.073	-0.452 *	—					
**WQ1**	0.036	0.160	0.058	0.174	-0.000	0.083	0.099	—				
**WQ2**	0.242	0.228	0.288	-0.018	0.167	0.190	0.144	0.360 *	—			
**WQ3**	0.211	0.092	0.129	-0.301	0.032	0.255	-0.350	0.576 ***	0.109	—		
**AQ**	-0.148	0.251	0.105	-0.143	0.219	-0.015	-0.093	-0.245	0.085	-0.257	—	
**SSQ**	0.368 *	-0.329	0.361	-0.352	-0.263	0.399 *	-0.101	-0.062	0.015	0.112	-0.246	—

Note. * p < .05, ** p < .01, *** p < .001. T = travel times (T1-T2), D = distance travelled (T1-T2), N = neutral, F = fear, A = Anger, WQ1 = orientation and navigation, WQ2 = spatial anxiety, WQ3 = distance estimation, AQ = Autism Spectrum Quotient scale, SSQ = Simulator Sickness Questionnaire.

Table SM4.6. Male participants. Partial correlation table including the experimental dependent variables and the exploratory variables of the main study.

Correlation - Pearson's r

	AGE	T-N	T-F	T-A	D-N	D-F	D-A	WQ1	WQ2	WQ3	AQ	SSQ
**AGE**	—											
**T-N**	0.229	—										
**T-F**	-0.105	-0.049	—									
**T-A**	-0.325	0.168	0.642 ***	—								
**D-N**	0.197	0.929 ***	-0.203	0.110	—							
**D-F**	-0.161	-0.000	0.972 ***	0.726 ***	-0.152	—						
**D-A**	-0.180	0.062	0.640 ***	0.806 ***	0.022	0.734 ***	—					
**WQ1**	0.017	0.313	-0.465 *	0.054	0.378	-0.359	-0.014	—				
**WQ2**	-0.137	0.544 **	-0.317	0.146	0.499 *	-0.201	0.040	0.676 ***	—			
**WQ3**	-0.140	-0.021	-0.652 ***	-0.188	-0.002	-0.520 **	-0.184	0.747 ***	0.625 **	—		
**AQ**	0.012	-0.339	0.110	0.068	-0.344	0.023	-0.165	-0.098	-0.316	-0.131	—	
**SSQ**	0.191	-0.272	0.092	0.021	-0.275	0.102	0.104	-0.293	-0.184	-0.061	0.194	—

Note. * $p < .05$, ** $p < .01$, *** $p < .001$. T = travel times (T1-T2), D = distance travelled (T1-T2),N = neutral, F = fear, A = Anger, WQ1 = orientation and navigation, WQ2 = spatial anxiety, WQ3 = distance estimation, AQ = Autism Spectrum Quotient scale, SSQ = Simulator Sickness Questionnaire.
